# Global incidence, mortality and temporal trends of cancer in children: A joinpoint regression analysis

**DOI:** 10.1002/cam4.5009

**Published:** 2022-07-13

**Authors:** Junjie Huang, Sze Chai Chan, Chun Ho Ngai, Veeleah Lok, Lin Zhang, Don Eliseo Lucero‐Prisno, Wanghong Xu, Zhi‐Jie Zheng, Edmar Elcarte, Mellissa Withers, Martin C. S. Wong, NCD Global Health Research Group, Association of Pacific Rim Universities (APRU)

**Affiliations:** ^1^ The Jockey Club School of Public Health and Primary Care, Faculty of Medicine Chinese University of Hong Kong Hong Kong SAR China; ^2^ Department of Global Public Health, Karolinska Institute Karolinska University Hospital Stockholm Sweden; ^3^ School of Population and Global Health The University of Melbourne Victoria Australia; ^4^ School of Public Health The Chinese Academy of Medical Sciences and Peking Union Medical College Beijing China; ^5^ Department of Global Health and Development London School of Hygiene and Tropical Medicine London UK; ^6^ School of Public Health Fudan University Shanghai China; ^7^ Department of Global Health, School of Public Health Peking University Beijing China; ^8^ University of the Philippines Manila the Philippines; ^9^ Department of Population and Public Health Sciences, Institute for Global Health University of Southern California Los Angeles California USA

**Keywords:** cancer, childhood, epidemiology, incidence, mortality, temporal trend

## Abstract

**Background/Methods:**

*The Cancer Incidence in Five Continents Time Trends*, *Nordic Cancer Registries*, *Surveillance, Epidemiology and End Results*, *WHO Mortality databases* were assessed to extract the Age‐Standardised Rates (ASR) of cancer incidence and mortality among children aged 0–14 years old. By using the ASRs, the country‐specific Average Annual Percentage Change (AAPC) and its corresponding 95% confidence interval (CI) were calculated to determine the epidemiological cancer trend.

**Results:**

In 2020, the highest incidence of childhood cancer was found in countries with higher Human Development Index (HDI) (ASR = 15.7), yet the highest mortality was found in countries with lower HDIs (ASR = 4.8). As for incidence, seven countries had positive AAPC among boys; Slovakia (AAPC_2001–2010_ = 4.98, 95% CI [1.66–8.40]), Ecuador (AAPC_2003–2012_ = 4.07, 95% CI [0.67–7.59]) and Thailand (AAPC_2003–2012_ = 3.69, 95% CI [0.37–7.11]) had the highest AAPC. Among girls, three countries had positive AAPC, which included Belarus (AAPC_2003–2012_ = 3.18, 95% CI [1.11, 5.29]), Canada (AAPC_2003–2012_ = 2.83, 95% CI [1.60, 4.07]) and Korea (AAPC_2003–2012_ = 1.76, 95% CI [0.23–3.32]). There was an overall decreasing trend of mortality. However, increased mortality was observed in two countries: Ecuador for boys (AAPC_2007–2016_ = 1.72, 95% CI [0.27–3.19]) and Austria for girls (AAPC_2008–2017_ = 4.11, 95% CI [0.38–7.98]).

**Conclusions:**

The largest mortality and mortality to incidence ratio of childhood cancer were found in low‐income countries. There was a substantial increasing trend of childhood cancer incidence, while overall its mortality has been decreasing over the past decade. More studies are needed to confirm the drivers behind these epidemiologic trends.

## Graphical abstract

This study aimed to explore the most up‐to‐date global burden and incidence/mortality temporal trends of cancer among children. It was found that the largest mortality and mortality to incidence ratio of childhood cancer were observed in low‐income countries; a substantial increasing trend in childhood cancer incidence was found, but its mortality has been decreasing generally over the past decade.

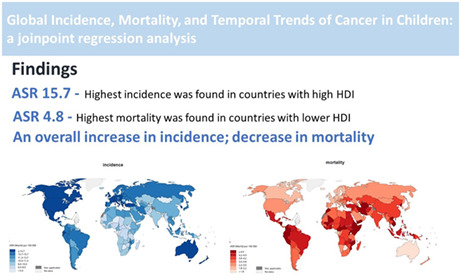

## 1 INTRODUCTION

Cancer is one of the leading causes of death among children.^1^ Despite childhood cancer is less common than cancer in adults, it has a significant impact on families and healthcare systems. Childhood cancers are often fatal without early diagnosis and treatment.^2^ Although globally there has been substantial socioeconomic development and improvement in healthcare systems, there are currently no lifestyle risk‐modification strategies and population‐based cancer screening programmes that are effective in improving childhood cancer survivorship.^3^ Therefore, improving outcomes for childhood cancer will require extensive planning to ensure adequate resource allocation and health system response.

The causes of childhood cancer remain unknown but may be related to genetic (gene mutation), inherited (passed on from first‐degree relatives), and environmental risk factors. Rare variation and non‐Mendelian inheritance, such as via maternal genetic effects or de novo germline mutations, may contribute to the risk of childhood cancer.^4^ Inherent risk factors, including low birth weight, older parental age and congenital anomalies, are consistently associated with the major types of childhood cancers.^5^ High dose ionising radiation and prior chemotherapy have also been confirmed as risk factors for childhood cancer.^6^, ^7^ However, the research is not as consistent for other potential environmental risk factors, including parental diet, maternal medication, caffeine and alcohol drinking.^6^

The examination of recent distribution and temporal trends of childhood cancer could be vital in the development and implementation of preventive strategies in different countries, as resource planning and allocation are important to reduce the burden of disease due to cancer. This study investigated the most updated incidence and mortality of childhood cancer on a global scale. It also examined the sex‐ and country‐specific temporal trends in incidence and mortality of all cancers among children.

## 2 METHODS

### 2.1 Sources of data

As this study focused on cancer temporal trends among children, data from individuals aged from 0 to 14 years old were used. To collect cancer incidence and mortality among children, different databases were accessed. The *Global Cancer Observatory (GLOBOCAN)* database, developed by the International Agency for Research on Cancer, World Health Organisation (IARC, WHO), has the most updated global cancer statistics and situation, which includes both incidence and mortality data for children in 183 countries.^8^ The Human Development Index (HDI) of a country was accessed from the United Nations. For trend analysis, the *Cancer Incidence in Five Continents Time Trends (CI5 Plus)*, the *Nordic Cancer Registries (NORDCAN)*, and the *Surveillance, Epidemiology and End Results Program (SEER)* were used to obtain cancer incidence from 48 countries from 1980 to 2017, time period varies among countries subject to data availability, detailed timeframe for each country can be found in Supplementary Table S1. The *CI5Plus* database is a comprehensive database that records cancer incidence rates and associated demographic characteristics for each cancer site, which makes comparisons across countries, sexes and age groups possible.^9^ The *NORDCAN* database, contains more detailed cancer‐related statistics for countries within the Nordic region.^10^, ^11^ The *SEER* programme, constructed by the National Cancer Institute of the United States Department of Health and Human Service, also has a detailed record of cancer statistics in the United States.^12^ As for mortality data, the WHO IARC mortality database records cancer‐caused deaths with demographic details, which was used to extract mortality rates.^13^ As this study included the temporal trends for incidence and mortality, the most recent mortality data were obtained from the *NORDCAN* and *SEER*. To ensure comparability, these countries and databases were selected only if (1) they contained population‐based registries of cancer incidence/mortality over a specific period; (2) cancer registries are developed by international guidelines to facilitate comparison; (3) the choice of registry is uniform throughout the period; (4) data of subgroup by age, sex and year are available; and (5) figures validated in previous publications examining the global trends of cancer burden. The database with the most updated data was used in case of overlapping data among databases. All extracted data were transformed into an age‐standardised rate (ASR) per 100,000 persons, which was adjusted according to the Segi‐Doll standard population that allowed the current study to compare incidence and mortality rates among different countries over time.^14^

### 2.2 Statistical analysis

Choropleth maps were generated to present the global incidence and mortality of childhood cancer. This study assessed temporal cancer trends among children over the last 10 years. The selection of a 10‐year timeframe considers both the sufficient use of available data and the effect of changes in registries, it is consistent with the practice of international publications in global trends of other cancers.^15^, ^16^ To analyse the temporal trend of cancer incidence and mortality rate, the measurement of Average Annual Percentage Change (AAPC) was utilised. The use of AAPC has been a common practice for determining the epidemiological trend of cancer^17^: it is a more suitable summary measure, in comparison to the Average Percentage Change (APC), to describe the trend of a fixed period, as it considers the lengths of the trends, while reporting the APC of a short time period may introduce uncertainty. For the calculation of AAPC, incidence and mortality ASRs of childhood cancer were collected from multiple databases for each country and then calculated by the joinpoint regression analysis. The current study utilised the joinpoint regression analysis software (Version 4.8.0.1 ‐ April 2020; Statistical Methodology and Applications Branch, Surveillance Research Program, National Cancer Institute), to calculate the AAPC and subsequently to determine the cancer incidence and mortality temporal trend. In order to calculate the AAPC, logarithmic transformation was performed on the retrieved ASRs from the previously mentioned databases. Then, a graph was plotted over the last 10 consecutive years, the time period used for trend analysis varied among countries and was subject to data availability, detailed information about the year selected can be found in supplementary Table S1. Over the 10 years, the joinpoint regression analysis software divided the data into segments and assigned a weighting for each segment according to each segment's length in proportion to the entire period, so that the AAPC could be calculated. The standard error of the AAPC was obtained from the binomial approximation, which was later used to compute the 95% Confidence Interval (CI). The AAPC was used to describe the temporal trend, with positive and negative AAPC representing an increasing and a decreasing trend in cancer incidence or mortality trend, respectively. The 95% CI determined the stability of the trend. To examine gender differences in incidence or mortality, the analysis included gender‐specific AAPC for individuals aged 0–14 years old. All tests with *p* values < 0.05 were considered statistically significant.

## 3 RESULTS

### 3.1 Global incidence of childhood cancer in 2020

**FIGURE 1:**
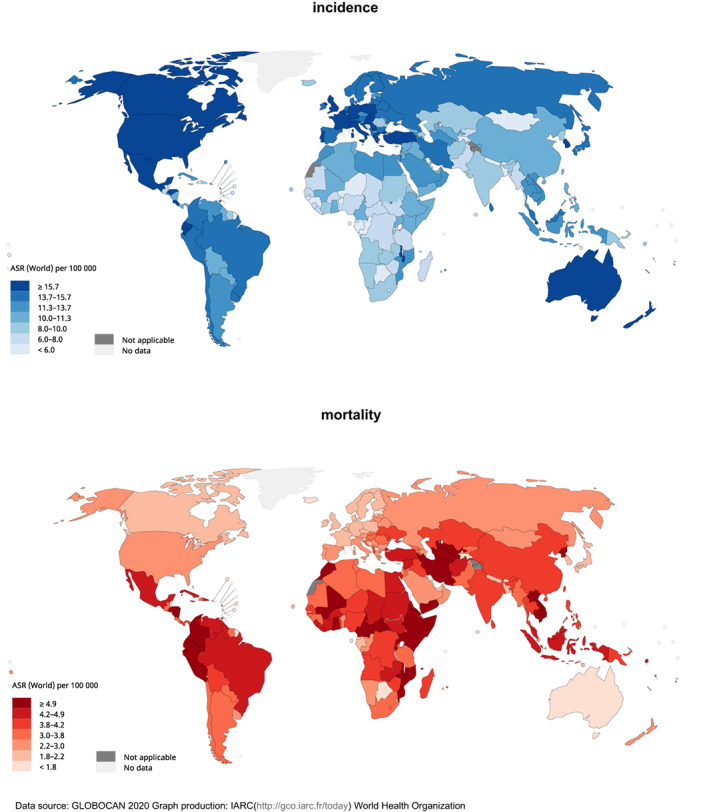
Global estimated burden of childhood cancer, both sexes, aged 0–14 years old, in 2020.

There were 206,362 newly reported cases of childhood cancer worldwide in 2020 (Figure 1). The global age‐standardised rate (ASR) of incidence was 10.5, with the highest incidence observed in North America (ASR = 17.3), Western Europe (ASR = 16.9), and Australia and New Zealand (ASR = 16.7). The impact of the Human Development Index (HDI) of a country on ASR of incidence was significant; populations in countries with very high HDIs (ASR = 15.7) had almost a 90% higher ASR than the population with medium (ASR = 8.5) or low HDIs (ASR = 8.5).

### 3.2 Global mortality of childhood cancer in 2020

In 2020, there were 80,104 child deaths related to cancer. The global ASR of mortality was 4.1 for both sexes. Notably, Eastern Africa (ASR = 5.6), Central America (ASR = 4.8) and South‐Eastern Asia (ASR = 4.5) reported the highest mortality of childhood cancer. The impact of HDI on ASR of mortality was remarkably different from that of incidence, as mortality rates in countries with low HDIs (ASR = 4.8) almost doubled that of those with very high HDIs (ASR = 2.6).

### 3.3 Temporal trends of childhood cancer

The incidence and mortality trends of childhood cancer for each country between 1980 and 2017 are shown in Figure S1, and the trend regression for the past 10 years is presented in Figure S2. Overall, more countries showed increasing trends than decreasing trends in incidence in both sexes, but especially for boys. As for mortality, more countries showed decreasing trends than increasing trends for both sexes. The AAPCs of the incidence and mortality trends of each country and their respective 95% CIs are listed in Table S2.

### 3.4 Trends of childhood cancer incidence

**FIGURE 2:**
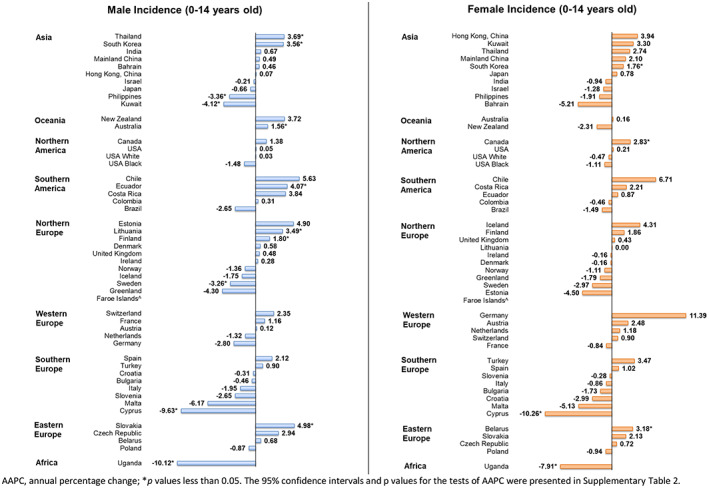
AAPC of childhood cancer incidence in subjects aged −14 years old.

Among boys, seven countries had increasing trends in incidence (Figure 2), with Slovakia reporting the highest AAPC (AAPC_2001–2010_ = 4.98, 95% CI [1.66, 8.40], *p* = 0.008), followed by Ecuador (AAPC_2003–2012_ = 4.07, 95% CI [0.67, 7.59], *p* = 0.024), and Thailand (AAPC_2003–2012_ = 3.69, 95% CI [0.37, 7.11], *p* = 0.033). On the contrary, five countries showed decreasing trends in incidence, with Uganda (AAPC_2003–2012_ = −10.12, 95% CI [−12.45, −7.73], *p* < 0.001), Cyprus (AAPC_2003–2012_ = −9.63, 95% CI [−13.22, −5.89], *p* < 0.001), and Kuwait (AAPC_2003–2012_ = −4.12, 95% CI [−7.00, −1.15], *p* = 0.013) reporting the largest decreases.

For girls, three countries had increasing trends, which included Belarus (AAPC_2003–2012_ = 3.18, 95% CI [1.11, 5.29], *p* = 0.007), Canada (AAPC_2003–2012_ = 2.83, 95% CI [1.60, 4.07], *p* < 0.001), and Korea (AAPC_2003–2012_ = 1.76, 95% CI [0.23, 3.32], *p* = 0.029). On the other hand, only two countries had decreasing trends: Cyprus (AAPC_2003–2012_ = −10.26, 95% CI [−18.39, −1.32], *p* = 0.030) and Uganda (AAPC_2003–2012_ = −7.91, 95% CI [−10.61, −5.12], *p* < 0.001). Overall, Korea was the only country that had increasing trends in both sexes. Conversely, Uganda and Cyprus were the only countries with decreasing trends in both sexes.

### 3.5 Trends of childhood cancer mortality

**FIGURE 3:**
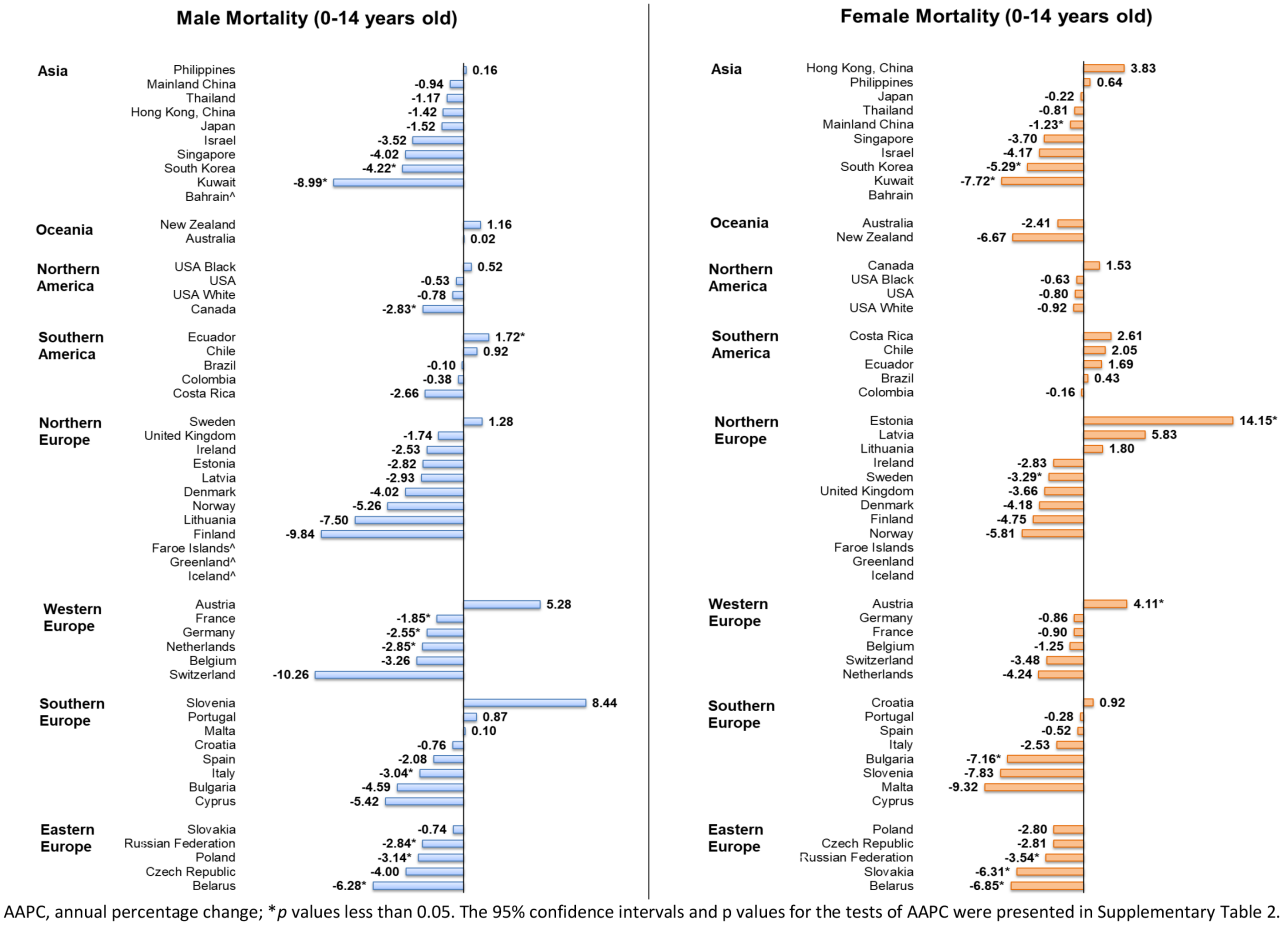
AAPC of childhood cancer mortality in subjects aged −14 years old.

Among boys, Ecuador (AAPC_2007–2016_ = 1.72, 95% CI [0.27, 3.19], *p* = 0.025) was the only country with a significant increasing trend of mortality (Figure 3). By contrast, 19 countries reported significant decreasing trends, with Kuwait (AAPC_2005‐2014_ = −8.99, 95% CI [−16.22, −1.13], *p* = 0.030), Belarus (AAPC_1986–1995_ = −6.28, 95% CI [−9.79, −2.63], *p* = 0.004), and Korea (AAPC_2007–2016_ = −4.22, 95% CI [−6.78, −1.60], *p* < 0.001) showing the most noteworthy decreases.

Among girls, Estonia (AAPC_2007–2016_ = 14.15, 95% CI [2.27, 27.41], *p* = 0.024) and Austria (AAPC_2008–2017_ = 4.11, 95% CI [0.38–7.98], *p* = 0.034) were the only countries with a significant increasing trend. On the contrary, eight countries reported significant decreases. The largest decreases were reported in Kuwait (AAPC_2005–2014_ = −7.72, 95% CI [−14.70, −0.16], *p* = 0.046), Bulgaria (AAPC_2006–2015_ = −7.16, 95% CI [−12.19, −1.85], *p* = 0.015), and Belarus (AAPC_1986–1995_ = −6.85, 95% CI [−9.72, −3.89], *p* < 0.001). Overall, Kuwait, Belarus, Korea and the Russian Federation were the four countries in which significant decreases in mortality rate were reported in both sexes.

## 4 DISCUSSION

### 4.1 Summary of major findings

This study evaluated the most updated cancer incidence, mortality and epidemiologic trends among children by sex, country and region using figures obtained from global cancer registries and databases. There were some major findings: (1) leukaemia, brain tumours and non‐Hodgkin lymphoma were the most common cancers and the leading causes of cancer deaths among children; (2) the highest incidence of childhood cancer was found in countries with higher HDIs, yet the highest mortality was found in countries with lower HDIs; (3) more countries were showing increasing trends than decreasing trends in incidence of childhood cancer, particularly in boys; while mortality has been decreasing consistently.

The characteristics of childhood cancer are fundamentally distinct from the cancers detected in adults. The cancer mortality among children remains high in developing countries, which is substantially different from the cancer patterns in adults.^18^ The cancer death among adults is usually heavier in wealthy countries, probably due to higher proportions of older populations and the prevalence of lifestyle and metabolic risk factors in these countries.^19^ Cancers burden among children are usually not effectively reduced by screening or risk modification as with cancers in adults.^20^ As a result, childhood cancers often progress rapidly and are fatal without appropriate and timely diagnosis and treatment.^21^ Therefore, well‐functioning healthcare systems with enough capacity for early detection and effective treatment are needed to improve the survival rates for cancer among children.

We found the highest incidence of childhood cancer occurs in countries with higher HDIs, yet the highest mortality is found in countries with lower HDIs. Although there is variation in the prevalence of environmental risk factors between geographical locations, and much to explore in terms of genetic differences between ethnicities, these factors alone might not explain the observed variation in the incidence of cancer among children by HDI.^22^ Previous studies noted that the low incidence found in low‐HDI countries could possibly be attributable to lower diagnosis, while higher exposure to aromatic cyclic hydrocarbon and nitrogen dioxide in high‐HDI populations might have contributed to the higher incidence.^22^ Despite advances and development in early detection and treatment of childhood cancer that have occurred in recent decades, differences in mortality between developed and developing countries persist.^1^, ^23^, ^24^ The higher mortality of childhood cancer observed in low‐ and middle‐income countries (LMICs) is probably attributable to several factors: specialised paediatric services are only provided in major cities, and health systems are not equipped to provide adequate care for childhood cancer patients in resource‐constrained settings.^25^, ^26^ Even in wealthy countries, limited access to care for children living in rural areas can have limited access to care, delay diagnosis and treatment disruption with unfavourable effects that undermine the achievement of the optimal prognosis.^27^, ^28^

The reasons for the increasing incidence of childhood cancer observed remain unclear but may be related to fertility treatment, increasing parental age and maternal use of hormonal contraception.^20^, ^29^ In contrast, this study found that mortality due to childhood cancer has consistently decreased, which mirrors results from a study that found that incidence of childhood cancer increased on average by 0.5% per year in Europe.^30^ Despite its general increasing trend of incidence over time, the observed decreases in mortality are likely attributable to better prognosis resulting from improved treatment and supportive care for childhood cancer. A global study found that survival rates for children increased steadily for leukaemia, central nervous system (CNS) tumours and non‐CNS solid tumours from 2000 to 2014.^31^ Reduced cancer mortality rates among adults may likely result from screening programmes or lifestyle modifications while decreases in childhood cancer mortality are likely to be due to improved early diagnosis and advancements in treatment protocols.

## 5 LIMITATIONS

There are several limitations to this study. The actual incidence and mortality of childhood cancer in developing countries might be higher, as due to underdevelopment of the mechanism of cancer reporting, while there might be overestimation for some countries because their figures were represented by cancer registries in major cities. Second, direct comparisons between countries may be imprecise, as cancer registration protocols may have changed over time. However, comparison of incidence and mortality over time within the same country would not be affected. Lastly, there this study did not examine trends of the different histological subtypes of cancer among children due to limited data available. As geographical distribution, risk factors and epidemic trends could vary by different subtypes of childhood cancer, this information might bear important implications for cancer prevention. Moreover, the GLOBOCAN and International Classification of Childhood Cancer, Third Edition (ICCC‐3) has various disparities in terms of classification system, the inclusion of registries and the participation rates of lower‐income countries.^3^ Therefore, comparison made between studies using different databases should be conducted with caution.

## 6 CONCLUSIONS

The largest mortality and mortality‐to‐incidence ratio of childhood cancer were found in countries with lower HDI. This could be due to the suboptimal resources available for early detection, treatment and surveillance for children with cancer in these regions compared with wealthy countries, highlighting the need for a better resource allocation and an improvement in healthcare facilities in those LMICs. A substantial increasing trend of childhood cancer incidence was noted, particularly among boys from Slovakia, Ecuador and Thailand, indicating the potential need for lifestyle modification. However, the mortality rates for childhood cancer overall have decreased in the past decade, likely due to the recent improvement of health facilities, novel therapeutic strategies and targeted drugs for childhood cancer. It is imperative to improve time to diagnosis, treatment, surveillance and patient quality of life for childhood cancer. More studies are needed to further confirm the drivers behind these epidemiologic trends and provide more insights into the specific aetiology and prognosis of childhood cancer by histological subtypes.

## AUTHOR CONTRIBUTIONS

JH and MCSW participated in the conception of the research ideas, study design, interpretation of the findings, writing of the first draft of the manuscript, and provided intellectual input to the translational aspects of the study. SCC, CHN and VL retrieved information from the relevant databases, performed the statistical analysis and presented the methodology and results. MW, LZ, DELP, WX, ZJZ and EE made critical revisions on the manuscripts and provided expert opinions on the implications of the study findings.

## CONFLICT OF INTEREST

The authors declare no conflict of interest.

## ETHICS APPROVAL

This study was approved by the Survey and Behavioural Research Ethics Committee, the Chinese University of Hong Kong (No. SBRE‐20‐332).

## Supporting information


Table S1

Table S2

Figure S1

Figure S2

## Data Availability

The data used for the analyses are publicly available from the World Health Organisation websites (https://gco.iarc.fr/; https://apps.who.int/gho/data/node.main).
