# Supplementary material for: Global incidence, mortality and temporal trends of cancer in children: A joinpoint regression analysis
Source: Cancer Med. 2022 Jul 13;12(2):1903–11. doi: 10.1002/cam4.5009 (PMC9883415; doi:10.1002/cam4.5009)
Supplement: Supplementary file 1 — Table S1 [file CAM4-12-1903-s002.docx]

**Supplementary Table 1.** Data sources for trend analysis

|  | Incidence  (line chart) | **Incidence**  **(trend analysis)** | | | Mortality  (line chart) | **Mortality**  **(trend analysis)** | |
| --- | --- | --- | --- | --- | --- | --- | --- |
| Australia | CI5 (1993-2012) | CI5 (2003-2012) | | | WHO (1980-2016) | WHO (2007-2016) | |
| Austria | CI5 (1998-2012) | CI5 (2003-2012) | | | WHO (1980-2017) | WHO (2008-2017) | |
| Bahrain | CI5 (1998-2012) | CI5 (2003-2012) | | | n/a | n/a | |
| Belarus | CI5 (1983-2012) | CI5 (2003-2012) | | | WHO (1981-2014) | WHO (1986-1995) | |
| Belgium | n/a | n/a | | | WHO (1980-2016) | WHO (2007-2016) | |
| Brazil | CI5 (1993-2012)1 | CI5 (2003-2012)1 | | | WHO (1996-2016) | WHO (2007-2016) | |
| Bulgaria | CI5 (1998-2012) | CI5 (2003-2012) | | | WHO (1980-2015) | WHO (2006-2015) | |
| Canada | CI5 (1983-2012)2 | CI5 (2003-2012)2 | | | WHO (1980-2015) | WHO (2006-2015) | |
| Chile | CI5 (1998-2012)3 | CI5 (2003-2012)3 | | | WHO (1980-2015) | WHO (2006-2015) | |
| China | CI5 (1998-2012)4 | CI5 (2003-2012)4 | | | WHO (1987-2000) | WHO (1991-2000) | |
| Colombia | CI5 (1983-2012)5 | CI5 (2003-2012)5 | | | WHO (1984-2015) | WHO (2006-2015) | |
| Costa Rica | CI5 (1982-2011) | CI5 (2002-2011) | | | WHO (1980-2014) | WHO (2005-2014) | |
| Croatia | CI5 (1988-2012) | CI5 (2003-2012) | | | WHO (1985-2016) | WHO (2007-2016) | |
| Cyprus | CI5 (1998-2012) | CI5 (2003-2012) | | | WHO (2004-2018) | Male: WHO (2006-2015)  Female: n/a | |
| Czech Republic | CI5 (1983-2012) | CI5 (2003-2012) | | | WHO (1986-2017) | WHO (2008-2017) | |
| Denmark | NORDCAN (1980-2016) | NORDCAN (2007-2016) | | | NORDCAN (1980-2016) | NORDCAN (2007-2016) | |
| Ecuador | CI5 (1985-2012)6 | | CI5 (2003-2012)6 | | WHO (1980-2016) | | WHO (2007-2016) |
| Estonia | CI5 (1983-2012) | | CI5 (2003-2012) | | WHO (1981-2016) | | WHO (2007-2016) |
| Faroe Islands | NORDCAN (1980-2015) | | n/a | | NORDCAN (1983-2013) | | n/a |
| Finland | NORDCAN (1980-2016) | | NORDCAN (2007-2016) | | NORDCAN (1980-2016) | | NORDCAN (2007-2016) |
| France | CI5 (1998-2011)7 | | CI5 (2002-2011)7 | | WHO (1980-2015) | | WHO (2006-2015) |
| Germany | CI5 (1998-2012)8 | | CI5 (2003-2012)8 | | WHO (1980-2016) | | WHO (2007-2016) |
| Greenland | NORDCAN (1980-2016) | | NORDCAN (1991-2000) | | NORDCAN (1983-2016) | | n/a |
| Hong Kong, SAR, China | CI5 (1983-2012) | | CI5 (2003-2012) | | WHO (1980-2016) | | WHO (2007-2016) |
| Iceland | NORDCAN (1980-2016) | | NORDCAN (2007-2016) | | NORDCAN (1980-2016) | | n/a |
| India | CI5(1983-2012)9 | | CI5(2003-2012)9 | | n/a | | n/a |
| Ireland | CI5 (1994-2012) | | CI5 (2003-2012) | | WHO (1980-2015) | | WHO (2006-2015) |
| Israel | CI5 (1980-2012) | | CI5 (2003-2012) | | WHO (1980-2016) | | WHO (2007-2016) |
| Italy | CI5 (1998-2010)10 | | CI5 (2001-2010)10 | | WHO (1980-2015) | | WHO (2006-2015) |
| Japan | CI5 (1998-2010)11 | | CI5 (2001-2010)11 | | WHO (1980-2016) | | WHO (2007-2016) |
| Korea (South) | CI5 (1999-2012)12 | | CI5 (2003-2012)12 | | WHO (1985-2016)13 | | WHO (2007-2016)13 |
| Kuwait | CI5 (1998-2012) | | CI5 (2003-2012) | | WHO (1980-2014) | | WHO (2005-2014) |
| Latvia | n/a | | n/a | | WHO (1980-2015) | | WHO (2006-2015) |
| Lithuania | CI5 (1988-2012) | | CI5 (2003-2012) | | WHO (1981-2015) | | WHO (2006-2015) |
| Malta | CI5 (1993-2012) | | CI5 (2003-2012) | | WHO (1980-2014) | | Male: WHO (2005-2014)  Female: n/a |
| Netherlands | CI5 (1989-2012) | | CI5 (2003-2012) | | WHO (1980-2016) | | WHO (2007-2016) |
| New Zealand | CI5 (1983-2012) | | CI5 (2003-2012) | | WHO (1980-2014) | | WHO (2005-2014) |
| Norway | NORDCAN (1980-2016) | | NORDCAN (2007-2016) | | NORDCAN (1980-2016) | | NORDCAN (2007-2016) |
| Philippines | CI5 (1983-2012)14 | | CI5 (2003-2012)14 | | WHO (1992-2011) | | WHO (1994-2003) |
| Poland | CI5 (1998-2012)15 | | CI5 (2003-2012)15 | | WHO (1980-2016) | | WHO (2007-2016) |
| Portugal | n/a | | n/a | | WHO (1980-2018) | | WHO (2009-2018) |
| Russian Federation | n/a | | n/a | | WHO (2005-2014) | | WHO (2005-2014) |
| Singapore | n/a | | n/a | | WHO (1980-2016) | | WHO (2007-2016) |
| Slovakia | CI5 (1980-2010) | | CI5 (2001-2010) | | WHO (1992-2014) | | WHO (2005-2014) |
| Slovenia | CI5 (1983-2012) | | CI5 (2003-2012) | | WHO (1985-2015) | | WHO (2006-2015) |
| Spain | CI5 (1993-2010)16 | | CI5 (2001-2010)16 | | WHO (1980-2016) | | WHO (2007-2016) |
| Sweden | NORDCAN (1980-2016) | | NORDCAN (2007-2016) | | NORDCAN (1980-2016) | | NORDCAN (2007-2016) |
| Switzerland | CI5 (1998-2012)17 | | CI5 (2003-2012)17 | | WHO (1980-2016) | | WHO (2007-2016) |
| Thailand | CI5 (1993-2012)18 | | | CI5 (2003-2012)18 | WHO (1980-2016) | | WHO (2007-2016) |
| Turkey | CI5 (1998-2012)19 | | | CI5 (2003-2012)19 | n/a | | n/a |
| Uganda | CI5 (1993-2012)20 | | | CI5 (2003-2012)20 | n/a | | n/a |
| United Kingdom | CI5 (1995-2012)21 | | | CI5 (2003-2012)21 | WHO (1980-2016) | | WHO (2007-2016) |
| USA | SEER (1980-2017) 22 | | | SEER (2008-2017) 22 | SEER (1980-2017) 22 | | SEER (2008-2017) 22 |

n/a” not available; CI5: Cancer Incidence in Five Continents V; NORDCAN: Nordic Cancer Registries’ SEER: USA: National Institutes of Health (NIH); WHO: World Health Organization

1. Brazil, Goiania
2. Canada (excl. Nunavut, Quebec and Yukon)
3. Chile, Valdivia
4. China (5 registries)
5. Colombia, Cali
6. Ecuador, Quito
7. France (9 registries)
8. Germany (2 registries)
9. India, Chennai
10. Italy (8 registries)
11. Japan (4 registries)
12. Korea (5 registries)
13. Republic of Korea
14. Philippines, Manila
15. Poland, Kielce
16. Spain (9 registries)
17. Switzerland (6 registries)
18. Thailand (4 registries)
19. Turkey (2 registries)
20. Uganda, Kampala
21. UK, England
22. USA, (8 registries)

**Reference:**

- 1. CI5: <http://ci5.iarc.fr/CI5plus/Pages/table1_sel.aspx>
  2. NORDCAN: <http://www-dep.iarc.fr/NORDCAN/english/frame.asp>
  3. SEER: <http://seer.cancer.gov/data/seerstat/>
  4. WHO: https://platform.who.int/mortality/themes/theme-details/topics/indicator-groups/indicator-group-details/MDB/testicular-cancer
