# Supplementary material for: Global incidence, mortality and temporal trends of cancer in children: A joinpoint regression analysis
Source: Cancer Med. 2022 Jul 13;12(2):1903–11. doi: 10.1002/cam4.5009 (PMC9883415; doi:10.1002/cam4.5009)
Supplement: Supplementary file 2 — Table S2 [file CAM4-12-1903-s003.docx]

**Supplementary Table 2.** Trend analysis of childhood cancer by country

a). Incidence trend in males aged 0-14 years

| **Region** | **AAPC** | **Lower CI** | **Upper CI** | **p-value** | **Significant** |
| --- | --- | --- | --- | --- | --- |
| ***Asia*** |  |  |  |  |  |
| Bahrain | 0.46 | -4.67 | 5.88 | 0.844 |  |
| China | 0.49 | -1.39 | 2.39 | 0.569 |  |
| Hong Kong, China | 0.07 | -3.12 | 3.36 | 0.963 |  |
| India | 0.67 | -3.09 | 4.57 | 0.697 |  |
| Israel | -0.21 | -2.33 | 1.95 | 0.826 |  |
| Japan | -0.66 | -4.50 | 3.33 | 0.707 |  |
| Korea | 3.56 | 1.86 | 5.30 | 0.001 | * |
| Kuwait | -4.12 | -7.00 | -1.15 | 0.013 | * |
| Philippines | -3.36 | -5.40 | -1.28 | 0.006 | * |
| Thailand | 3.69 | 0.37 | 7.11 | 0.033 | * |
| ***Oceania*** |  |  |  |  |  |
| Australia | 1.56 | 0.01 | 3.12 | 0.048 | * |
| New Zealand | 3.72 | -0.14 | 7.74 | 0.057 |  |
| ***Northern America*** |  |  |  |  |  |
| Canada | 1.38 | -0.06 | 2.85 | 0.059 |  |
| USA | 0.05 | -1.18 | 1.29 | 0.931 |  |
| USA Black | -1.48 | -5.17 | 2.36 | 0.394 |  |
| USA White | 0.03 | -1.49 | 1.57 | 0.967 |  |
| ***Southern America*** |  |  |  |  |  |
| Brazil | -2.65 | -7.01 | 1.92 | 0.214 |  |
| Chile | 5.63 | -5.97 | 18.67 | 0.309 |  |
| Colombia | 0.31 | -3.10 | 3.84 | 0.842 |  |
| Costa Rica | 3.84 | -0.10 | 7.95 | 0.055 |  |
| Ecuador | 4.07 | 0.67 | 7.59 | 0.024 | * |
| ***Northern Europe*** |  |  |  |  |  |
| Denmark | 0.58 | -3.69 | 5.05 | 0.766 |  |
| Estonia | 4.90 | -1.95 | 12.23 | 0.141 |  |
| Finland | 1.80 | 0.09 | 3.54 | 0.042 | * |
| Greenland | -4.30 | -15.71 | 8.65 | 0.447 |  |
| Iceland | -1.75 | -11.36 | 8.90 | 0.703 |  |
| Ireland | 0.28 | -2.46 | 3.10 | 0.820 |  |
| Lithuania | 3.49 | 0.56 | 6.50 | 0.019 | * |
| Norway | -1.36 | -4.81 | 2.22 | 0.402 |  |
| Sweden | -3.26 | -5.68 | -0.77 | 0.011 | * |
| United Kingdom | 0.48 | -0.56 | 1.52 | 0.319 |  |
| ***Western Europe*** |  |  |  |  |  |
| Austria | 0.12 | -4.36 | 4.81 | 0.960 |  |
| France | 1.16 | -1.58 | 3.98 | 0.360 |  |
| Germany | -2.80 | -9.02 | 3.84 | 0.350 |  |
| Netherlands | -1.32 | -2.77 | 0.14 | 0.070 |  |
| Switzerland | 2.35 | -2.95 | 7.93 | 0.343 |  |
| ***Southern Europe*** |  |  |  |  |  |
| Bulgaria | -0.46 | -4.44 | 3.69 | 0.801 |  |
| Croatia | -0.31 | -2.95 | 2.41 | 0.800 |  |
| Cyprus | -9.63 | -13.22 | -5.89 | 0.000 | * |
| Italy | -1.95 | -5.00 | 1.19 | 0.188 |  |
| Malta | -6.17 | -20.59 | 10.86 | 0.404 |  |
| Slovenia | -2.65 | -9.12 | 4.28 | 0.395 |  |
| Spain | 2.12 | -1.66 | 6.04 | 0.236 |  |
| Turkey | 0.90 | -1.33 | 3.18 | 0.383 |  |
| ***Eastern Europe*** |  |  |  |  |  |
| Belarus | 0.68 | -2.75 | 4.23 | 0.663 |  |
| Czech Republic | 2.94 | -2.52 | 8.72 | 0.255 |  |
| Poland | -0.87 | -8.88 | 7.84 | 0.817 |  |
| Slovakia | 4.98 | 1.66 | 8.40 | 0.008 | * |
| ***Africa*** |  |  |  |  |  |
| Uganda | -10.12 | -12.45 | -7.73 | <0.001 | * |

AAPC, annual percentage change; CI, confidence interval; * p values less than 0.05.

b). Incidence trend in females aged 0-14 years

| **Region** | **AAPC** | **Lower CI** | **Upper CI** | **p-value** | **Significant** |
| --- | --- | --- | --- | --- | --- |
| ***Asia*** |  |  |  |  |  |
| Bahrain | -5.21 | -15.55 | 6.39 | 0.316 |  |
| China | 2.10 | -1.07 | 5.37 | 0.167 |  |
| Hong Kong, China | 3.94 | -0.02 | 8.05 | 0.051 |  |
| India | -0.94 | -3.32 | 1.50 | 0.396 |  |
| Israel | -1.28 | -2.96 | 0.43 | 0.123 |  |
| Japan | 0.78 | -2.16 | 3.82 | 0.562 |  |
| Korea | 1.76 | 0.23 | 3.32 | 0.029 | * |
| Kuwait | 3.30 | -10.87 | 19.73 | 0.666 |  |
| Philippines | -1.91 | -4.90 | 1.17 | 0.188 |  |
| Thailand | 2.74 | -1.30 | 6.96 | 0.159 |  |
| ***Oceania*** |  |  |  |  |  |
| Australia | 0.16 | -1.20 | 1.54 | 0.795 |  |
| New Zealand | -2.31 | -6.57 | 2.14 | 0.260 |  |
| ***Northern America*** |  |  |  |  |  |
| Canada | 2.83 | 1.60 | 4.07 | 0.001 | * |
| USA | 0.21 | -0.42 | 0.84 | 0.470 |  |
| USA Black | -1.11 | -3.53 | 1.37 | 0.329 |  |
| USA White | -0.47 | -1.84 | 0.93 | 0.458 |  |
| ***Southern America*** |  |  |  |  |  |
| Brazil | -1.49 | -7.49 | 4.89 | 0.596 |  |
| Chile | 6.71 | -2.96 | 17.34 | 0.153 |  |
| Colombia | -0.46 | -3.07 | 2.21 | 0.696 |  |
| Costa Rica | 2.21 | -0.56 | 5.06 | 0.104 |  |
| Ecuador | 0.87 | -3.42 | 5.36 | 0.657 | * |
| ***Northern Europe*** |  |  |  |  |  |
| Denmark | -0.16 | -3.48 | 3.28 | 0.917 |  |
| Estonia | -4.50 | -13.76 | 5.75 | 0.328 |  |
| Finland | 1.86 | -0.22 | 3.98 | 0.073 |  |
| Iceland | 4.31 | -5.24 | 14.82 | 0.340 |  |
| Ireland | -0.16 | -3.32 | 3.11 | 0.914 |  |
| Lithuania | 0.00 | -4.08 | 4.26 | 0.999 |  |
| Norway | -1.11 | -4.46 | 2.36 | 0.478 |  |
| Sweden | -2.97 | -8.61 | 3.02 | 0.324 |  |
| United Kingdom | 0.43 | -0.96 | 1.85 | 0.499 |  |
| ***Western Europe*** |  |  |  |  |  |
| Austria | 2.48 | -1.67 | 6.80 | 0.209 |  |
| France | -0.84 | -3.13 | 1.51 | 0.430 |  |
| Germany | 11.39 | -0.36 | 24.53 | 0.058 |  |
| Netherlands | 1.18 | -1.01 | 3.42 | 0.253 |  |
| Switzerland | 0.90 | -3.27 | 5.25 | 0.637 |  |
| ***Southern Europe*** |  |  |  |  |  |
| Bulgaria | -1.73 | -4.30 | 0.90 | 0.166 |  |
| Croatia | -2.99 | -7.68 | 1.93 | 0.195 |  |
| Cyprus | -10.26 | -18.39 | -1.32 | 0.030 | * |
| Italy | -0.86 | -5.70 | 4.23 | 0.702 |  |
| Malta | -5.13 | -14.72 | 5.54 | 0.287 |  |
| Slovenia | -0.28 | -7.12 | 7.07 | 0.930 |  |
| Spain | 1.02 | -0.73 | 2.80 | 0.217 |  |
| Turkey | 3.47 | -0.26 | 7.33 | 0.065 |  |
| ***Eastern Europe*** |  |  |  |  |  |
| Belarus | 3.18 | 1.11 | 5.29 | 0.007 | * |
| Czech Republic | 0.72 | -3.88 | 5.54 | 0.764 |  |
| Poland | -0.94 | -6.78 | 5.27 | 0.729 |  |
| Slovakia | 2.13 | -0.30 | 4.62 | 0.079 |  |
| ***Africa*** |  |  |  |  |  |
| Uganda | -7.91 | -10.61 | -5.12 | <0.001 | * |

AAPC, annual percentage change; CI, confidence interval; * p values less than 0.05.

c). Mortality trend in males aged 0-14 years

| **Region** | **AAPC** | **Lower CI** | **Upper CI** | **p-value** | **Significant** |
| --- | --- | --- | --- | --- | --- |
| ***Asia*** |  |  |  |  |  |
| China | -0.94 | -2.09 | 0.23 | 0.102 |  |
| Hong Kong, China | -1.42 | -10.59 | 8.69 | 0.744 |  |
| Israel | -3.52 | -8.06 | 1.25 | 0.125 |  |
| Japan | -1.52 | -3.69 | 0.70 | 0.151 |  |
| Korea | -4.22 | -6.78 | -1.60 | 0.006 | * |
| Kuwait | -8.99 | -16.22 | -1.13 | 0.030 | * |
| Philippines | 0.16 | -1.12 | 1.46 | 0.780 |  |
| Singapore | -4.02 | -21.46 | 17.29 | 0.650 |  |
| Thailand | -1.17 | -3.52 | 1.25 | 0.295 |  |
| ***Oceania*** |  |  |  |  |  |
| Australia | 0.02 | -3.64 | 3.82 | 0.989 |  |
| New Zealand | 1.16 | -7.73 | 10.91 | 0.780 |  |
| ***Northern America*** |  |  |  |  |  |
| Canada | -2.83 | -5.56 | -0.01 | 0.049 | * |
| USA | -0.53 | -1.80 | 0.76 | 0.372 |  |
| USA Black | 0.52 | -2.40 | 3.54 | 0.693 |  |
| USA White | -0.78 | -1.83 | 0.29 | 0.132 |  |
| ***Southern America*** |  |  |  |  |  |
| Brazil | -0.10 | -1.03 | 0.83 | 0.803 |  |
| Chile | 0.92 | -1.57 | 3.47 | 0.423 |  |
| Colombia | -0.38 | -1.93 | 1.20 | 0.595 |  |
| Costa Rica | -2.66 | -7.91 | 2.90 | 0.295 |  |
| Ecuador | 1.72 | 0.27 | 3.19 | 0.025 | * |
| ***Northern Europe*** |  |  |  |  |  |
| Denmark | -4.02 | -13.01 | 5.90 | 0.364 |  |
| Estonia | -2.82 | -19.70 | 17.61 | 0.738 |  |
| Finland | -9.84 | -18.90 | 0.24 | 0.054 |  |
| Ireland | -2.53 | -9.99 | 5.55 | 0.479 |  |
| Latvia | -2.93 | -11.53 | 6.51 | 0.481 |  |
| Lithuania | -7.50 | -14.84 | 0.48 | 0.062 |  |
| Norway | -5.26 | -15.34 | 6.02 | 0.300 |  |
| Sweden | 1.28 | -7.85 | 11.31 | 0.765 |  |
| United Kingdom | -1.74 | -4.47 | 1.07 | 0.189 |  |
| ***Western Europe*** |  |  |  |  |  |
| Austria | 5.28 | -0.46 | 11.34 | 0.067 |  |
| Belgium | -3.26 | -6.80 | 0.43 | 0.075 |  |
| France | -1.85 | -3.35 | -0.33 | 0.023 | * |
| Germany | -2.55 | -4.63 | -0.42 | 0.025 | * |
| Netherlands | -2.85 | -5.11 | -0.54 | 0.022 | * |
| Switzerland | -10.26 | -20.68 | 1.53 | 0.086 |  |
| ***Southern Europe*** |  |  |  |  |  |
| Bulgaria | -4.59 | -9.84 | 0.96 | 0.092 |  |
| Croatia | -0.76 | -7.89 | 6.93 | 0.821 |  |
| Italy | -3.04 | -4.66 | -1.39 | 0.003 | * |
| Malta | 0.10 | -5.87 | 6.44 | 0.972 |  |
| Portugal | 0.87 | -3.70 | 5.65 | 0.678 |  |
| Slovenia | 8.44 | -3.78 | 22.20 | 0.157 |  |
| Spain | -2.08 | -5.39 | 1.35 | 0.232 |  |
| ***Eastern Europe*** |  |  |  |  |  |
| Belarus | -6.28 | -9.79 | -2.63 | 0.004 | * |
| Czech Republic | -4.00 | -8.01 | 0.19 | 0.059 |  |
| Poland | -3.14 | -5.98 | -0.21 | 0.039 | * |
| Russian Federation | -2.84 | -3.65 | -2.02 | <0.001 | * |
| Slovakia | -0.74 | -9.51 | 8.89 | 0.858 |  |
|  |  |  |  |  |  |

AAPC, annual percentage change; CI, confidence interval; * p values less than 0.05.

d) Mortality trend in females aged 0-14 years

| **Region** | **AAPC** | **Lower CI** | **Upper CI** | **p-value** | **Significant** |
| --- | --- | --- | --- | --- | --- |
| ***Asia*** |  |  |  |  |  |
| China | -1.23 | -2.16 | -0.29 | 0.017 | * |
| Hong Kong, China | 3.83 | -6.39 | 15.17 | 0.427 |  |
| Israel | -4.17 | -8.32 | 0.17 | 0.057 |  |
| Japan | -0.22 | -3.38 | 3.04 | 0.893 |  |
| Korea | -5.29 | -9.19 | -1.22 | 0.018 | * |
| Kuwait | -7.72 | -14.70 | -0.16 | 0.046 | * |
| Philippines | 0.64 | -1.16 | 2.46 | 0.439 |  |
| Singapore | -3.70 | -14.24 | 8.14 | 0.475 |  |
| Thailand | -0.81 | -2.34 | 0.74 | 0.262 |  |
| ***Oceania*** |  |  |  |  |  |
| Australia | -2.41 | -6.63 | 1.99 | 0.238 |  |
| New Zealand | -6.67 | -13.21 | 0.36 | 0.060 |  |
| ***Northern America*** |  |  |  |  |  |
| Canada | 1.53 | -2.43 | 5.65 | 0.405 |  |
| USA | -0.80 | -1.75 | 0.17 | 0.094 |  |
| USA Black | -0.63 | -3.24 | 2.06 | 0.602 |  |
| USA White | -0.92 | -2.18 | 0.35 | 0.133 |  |
| ***Southern America*** |  |  |  |  |  |
| Brazil | 0.43 | -0.60 | 1.46 | 0.365 |  |
| Chile | 2.05 | -0.87 | 5.05 | 0.146 |  |
| Colombia | -0.16 | -2.13 | 1.85 | 0.854 |  |
| Costa Rica | 2.61 | -3.20 | 8.77 | 0.338 |  |
| Ecuador | 1.69 | -0.80 | 4.25 | 0.157 |  |
| ***Northern Europe*** |  |  |  |  |  |
| Denmark | -4.18 | -13.09 | 5.64 | 0.342 |  |
| Estonia | 14.15 | 2.27 | 27.41 | 0.024 | * |
| Finland | -4.75 | -10.26 | 1.10 | 0.096 |  |
| Ireland | -2.83 | -11.41 | 6.57 | 0.494 |  |
| Latvia | 5.83 | -5.06 | 17.97 | 0.263 |  |
| Lithuania | 1.80 | -8.64 | 13.43 | 0.714 |  |
| Norway | -5.81 | -18.47 | 8.80 | 0.366 |  |
| Sweden | -3.29 | -6.85 | 0.41 | 0.074 |  |
| United Kingdom | -3.66 | -6.30 | -0.95 | 0.015 | * |
| ***Western Europe*** |  |  |  |  |  |
| Austria | 4.11 | 0.38 | 7.98 | 0.034 | * |
| Belgium | -1.25 | -5.99 | 3.73 | 0.571 |  |
| France | -0.90 | -3.01 | 1.25 | 0.361 |  |
| Germany | -0.86 | -2.56 | 0.88 | 0.287 |  |
| Netherlands | -4.24 | -8.32 | 0.03 | 0.051 |  |
| Switzerland | -3.48 | -13.60 | 7.83 | 0.482 |  |
| ***Southern Europe*** |  |  |  |  |  |
| Bulgaria | -7.16 | -12.19 | -1.85 | 0.015 | * |
| Croatia | 0.92 | -7.11 | 9.65 | 0.805 |  |
| Italy | -2.53 | -6.55 | 1.67 | 0.199 |  |
| Malta | -2.34 | -9.32 | 5.18 | 0.483 |  |
| Portugal | -0.28 | -5.49 | 5.22 | 0.908 |  |
| Slovenia | -7.83 | -23.42 | 10.94 | 0.389 |  |
| Spain | -0.52 | -4.27 | 3.39 | 0.763 |  |
| ***Eastern Europe*** |  |  |  |  |  |
| Belarus | -6.85 | -9.72 | -3.89 | 0.001 | * |
| Czech Republic | -2.81 | -9.13 | 3.94 | 0.356 |  |
| Poland | -2.80 | -6.06 | 0.58 | 0.104 |  |
| Russian Federation | -3.54 | -5.16 | -1.89 | 0.001 | * |
| Slovakia | -6.31 | -10.66 | -1.75 | 0.013 | * |
|  |  |  |  |  |  |

AAPC, annual percentage change; CI, confidence interval; * p values less than 0.05.
