# Supplementary material for: Global incidence, mortality and temporal trends of cancer in children: A joinpoint regression analysis
Source: Cancer Med. 2022 Jul 13;12(2):1903–11. doi: 10.1002/cam4.5009 (PMC9883415; doi:10.1002/cam4.5009)
Supplement: Supplementary file 3 — Figure S1 [file CAM4-12-1903-s001.docx]

**Supplementary Figure 1:** The plots of incidence and mortality trends for each country

**Male**

| **Asia** | | |
| --- | --- | --- |
|  |  |  |
|  |  |  |
|  |  |  |
|  |  |  |

| **Oceania** | | |
| --- | --- | --- |
|  |  |  |
| **Northern America** | | |
|  |  |  |
|  |  |  |
| **Southern America** | | |
|  |  |  |
|  |  |  |
| **Northern Europe** | | |
|  |  |  |
|  |  |  |
|  |  |  |
|  |  |  |
| **Western Europe** | | |
|  |  |  |
|  |  |  |
|  |  |  |
| **Southern Europe** | | |
|  |  |  |
|  |  |  |
|  |  |  |
| **Eastern Europe** | | |
|  |  |  |
|  |  |  |
| **Africa** | | |
|  |  |  |

**Female**

| **Asia** | | |
| --- | --- | --- |
|  |  |  |
|  |  |  |
|  |  |  |
|  |  |  |

| **Oceania** | | |
| --- | --- | --- |
|  |  |  |
| **Northern America** | | |
|  |  |  |
|  |  |  |
| **Southern America** | | |
|  |  |  |
|  |  |  |
| **Northern Europe** | | |
|  |  |  |
|  |  |  |
|  |  |  |
|  |  |  |
| **Western Europe** | | |
|  |  |  |
|  |  |  |
|  |  |  |
| **Southern Europe** | | |
|  |  |  |
|  |  |  |
|  |  |  |
| **Eastern Europe** | | |
|  |  |  |
|  |  |  |
| **Africa** | | |
|  |  |  |
