# Supplementary material for: Global incidence, mortality and temporal trends of cancer in children: A joinpoint regression analysis
Source: Cancer Med. 2022 Jul 13;12(2):1903–11. doi: 10.1002/cam4.5009 (PMC9883415; doi:10.1002/cam4.5009)
Supplement: Supplementary file 4 — Figure S2 [file CAM4-12-1903-s004.docx]

**Supplementary Figure 2:** Results of joinpoint regression for individual countries

a.) Incidence male aged 0-14

| **Asia** | |
| --- | --- |
| 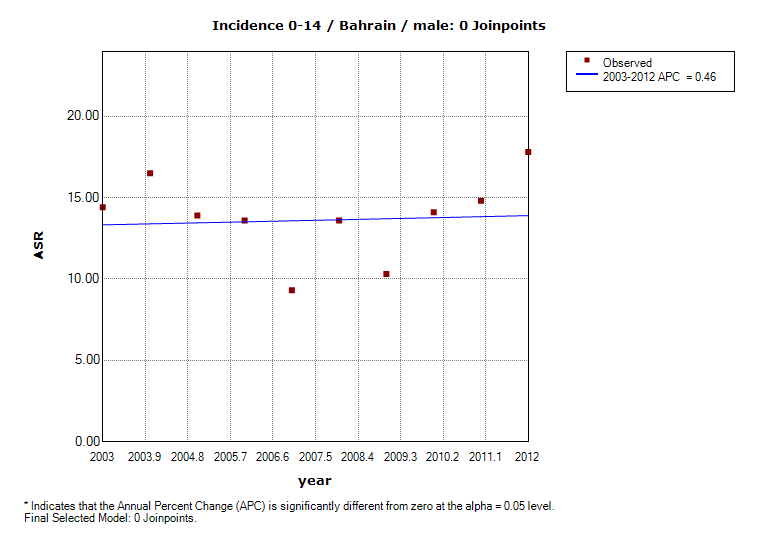 | 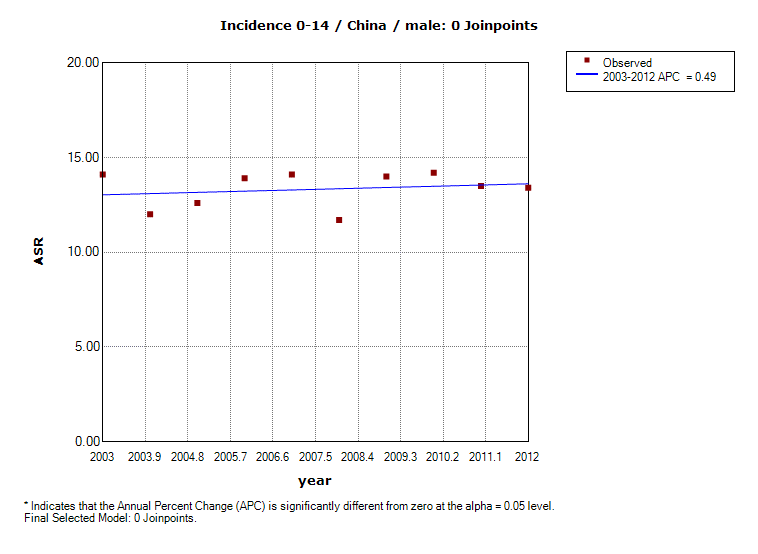 |
| 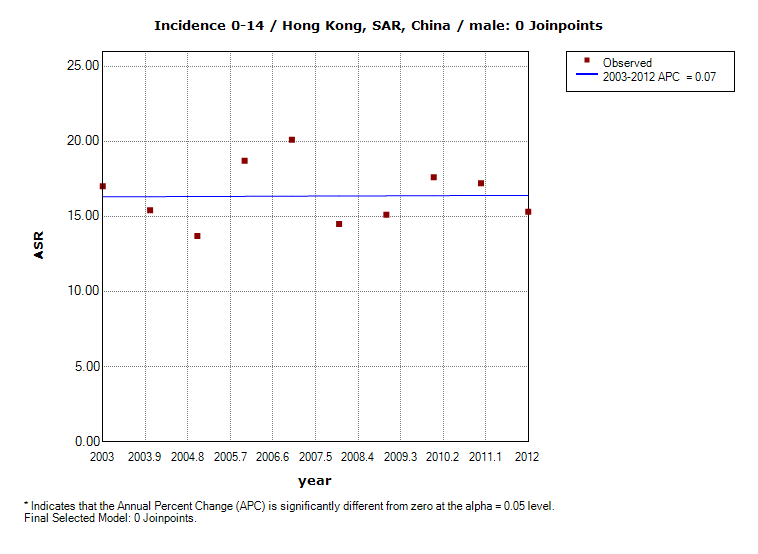 | 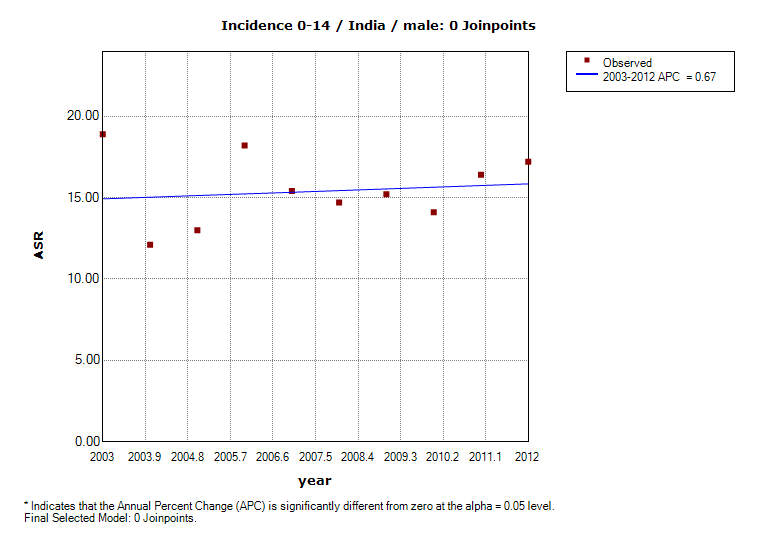 |
| 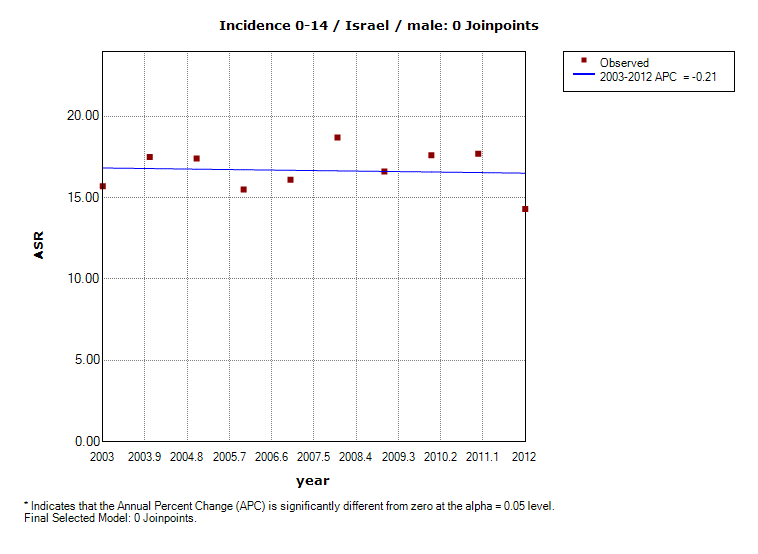 | 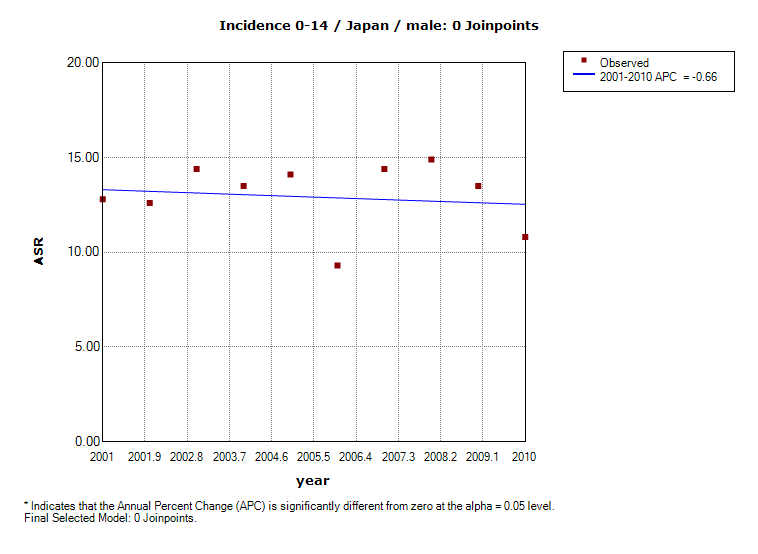 |
| 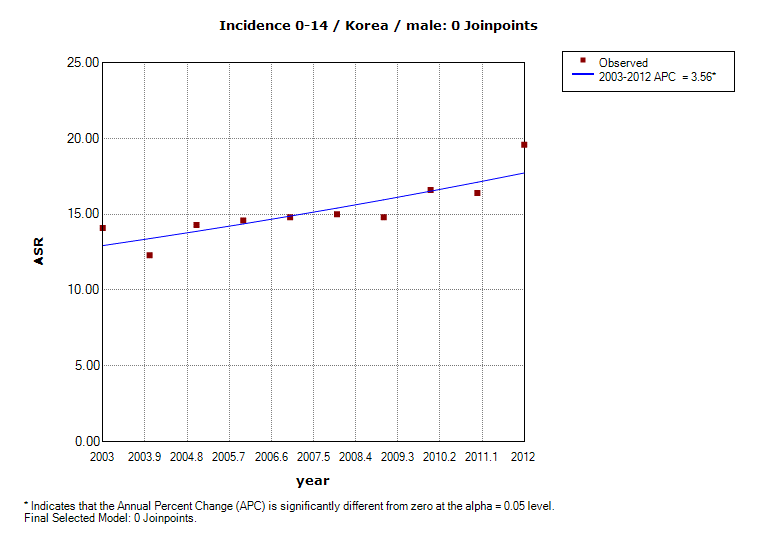 | 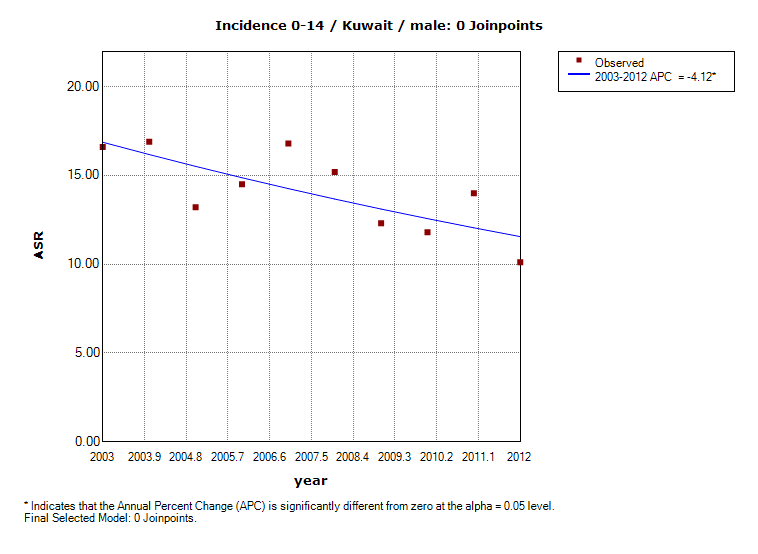 |
| 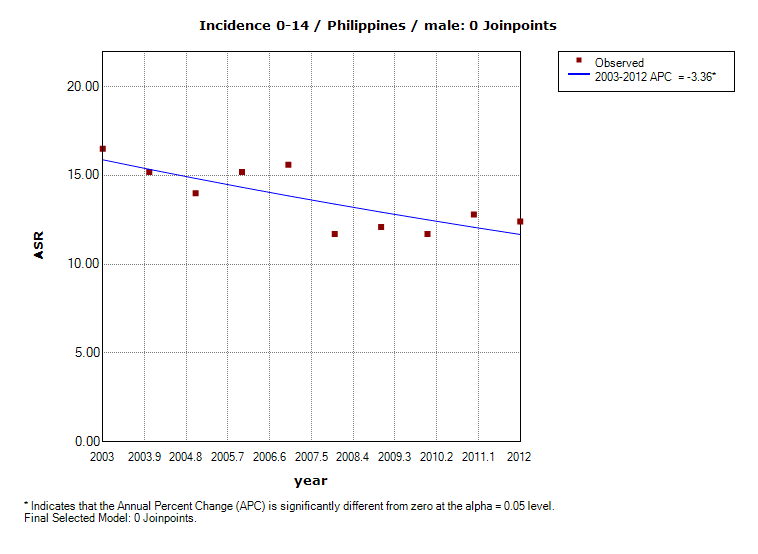 | 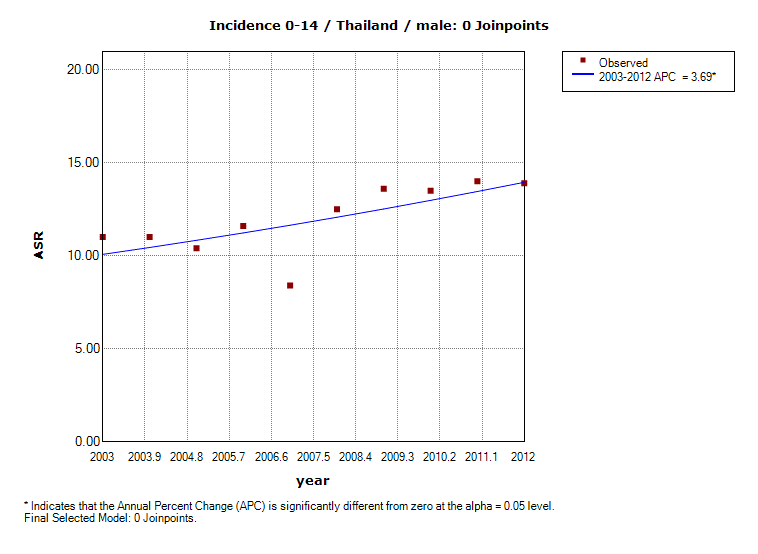 |
| **Oceania** | |
| 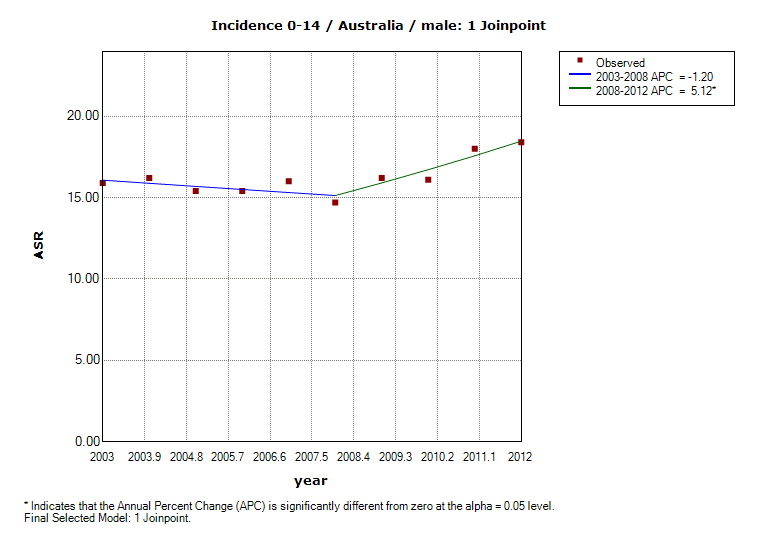 | 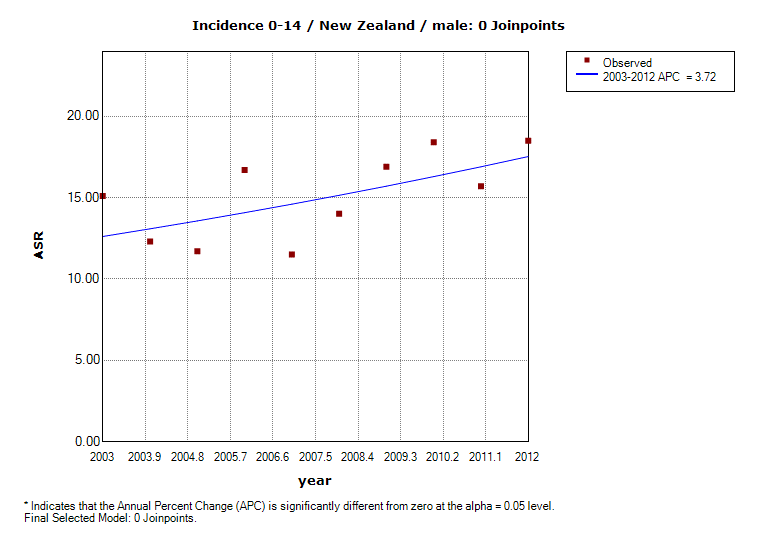 |

| **Northern America** | |
| --- | --- |
| 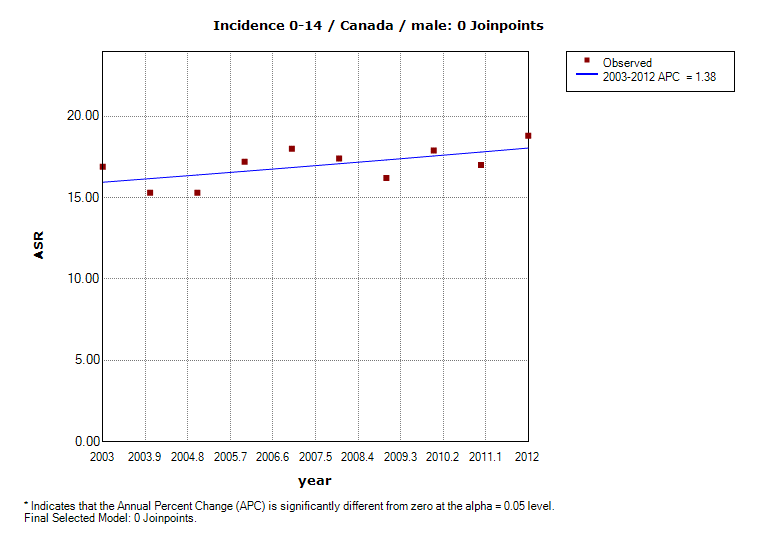 | 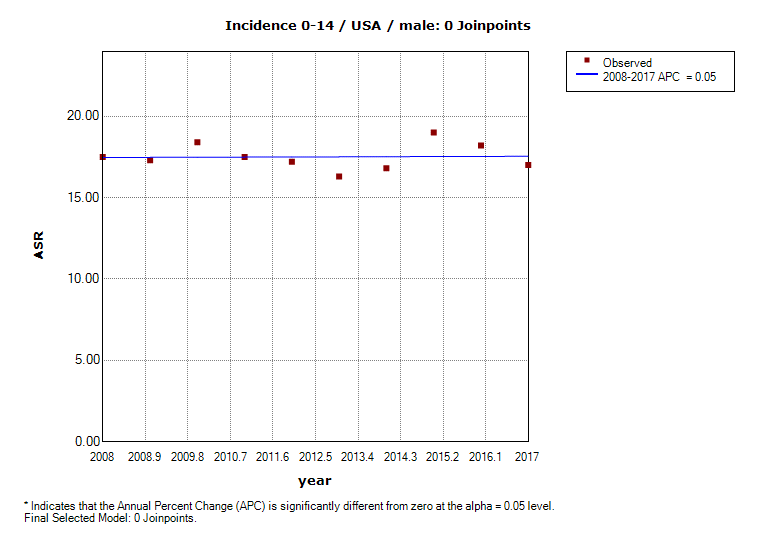 |
| 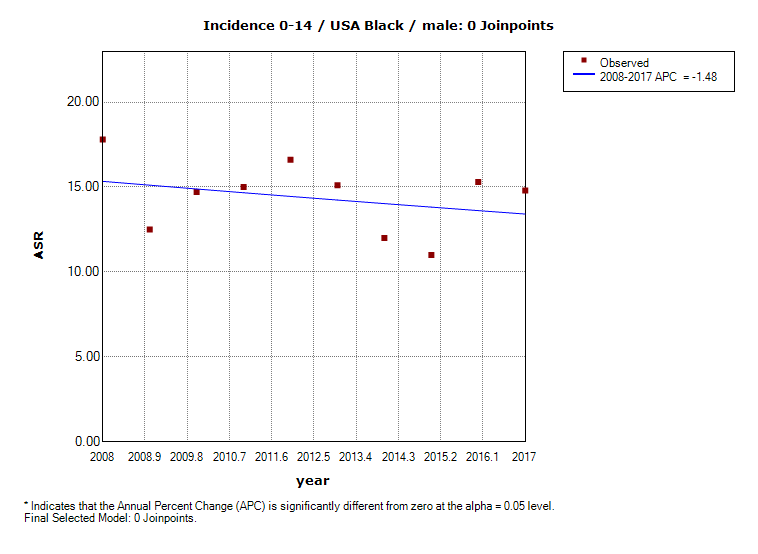 | 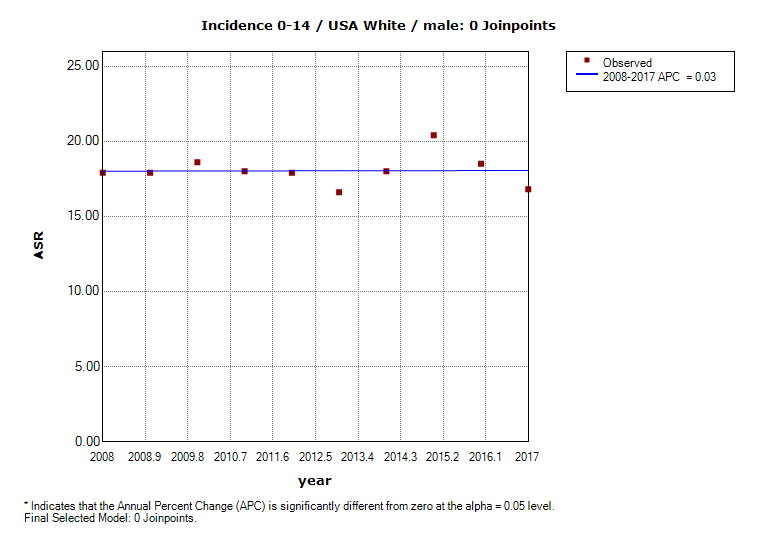 |
|  | |
| 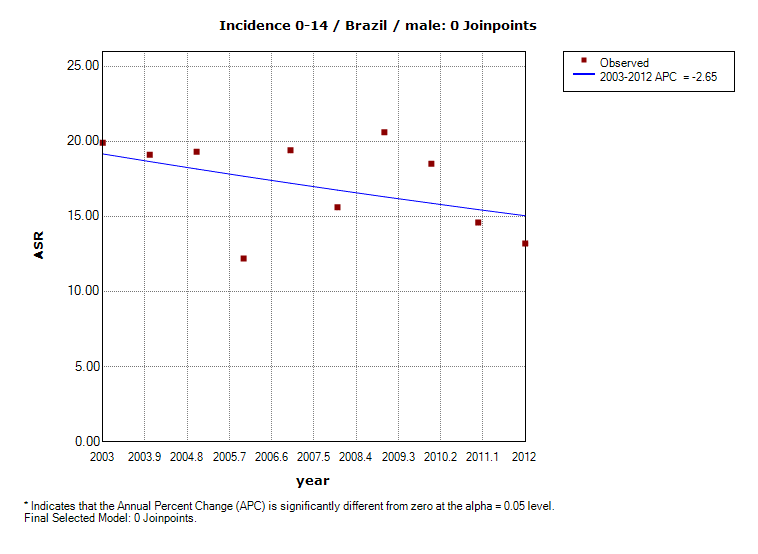 | 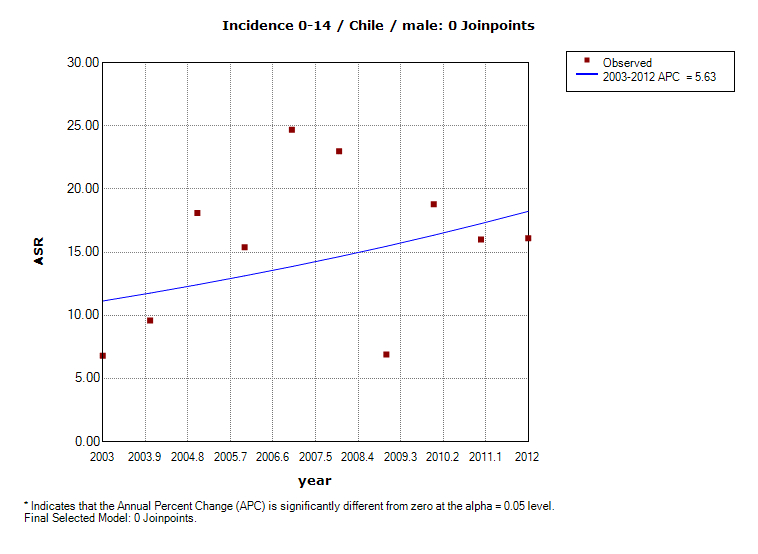 |
| 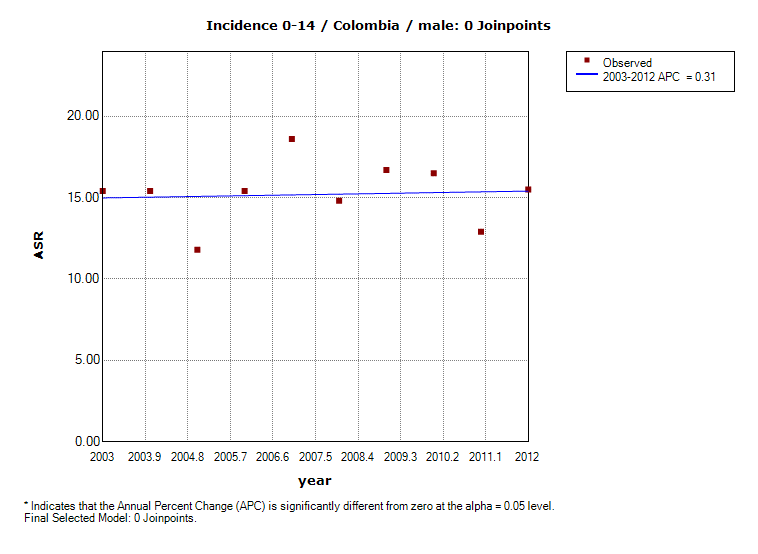 | 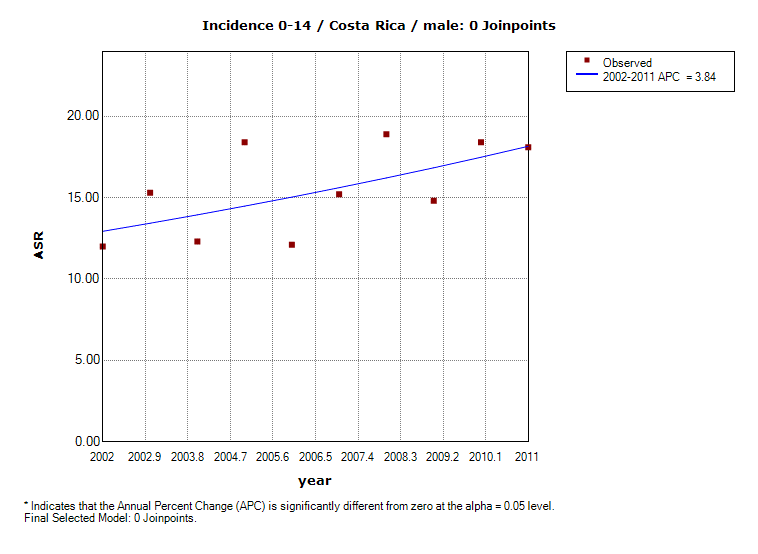 |
| 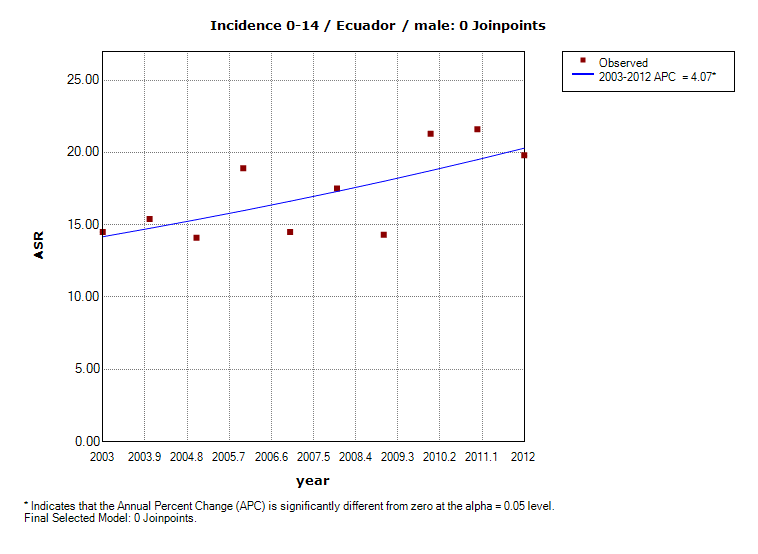 |  |
| **Northern Europe** | |
| 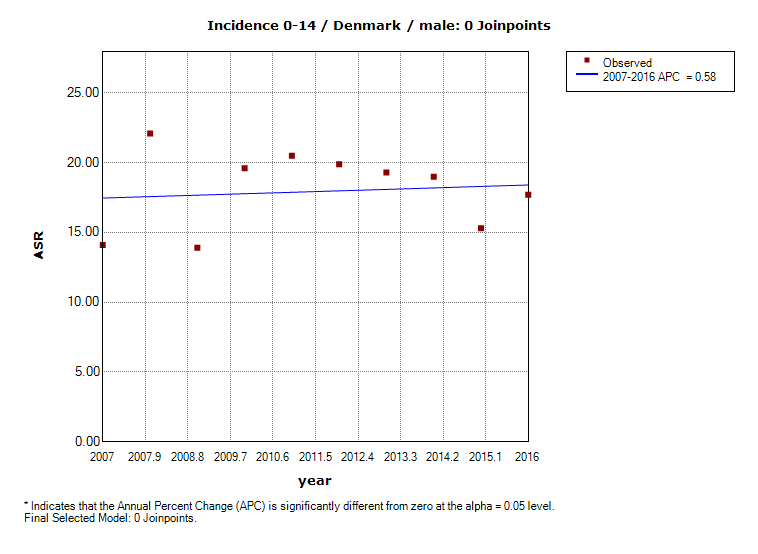 | 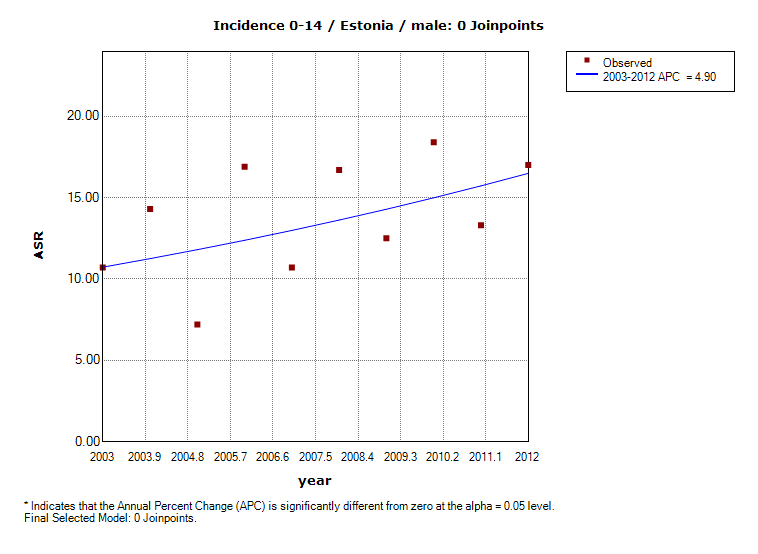 |
| 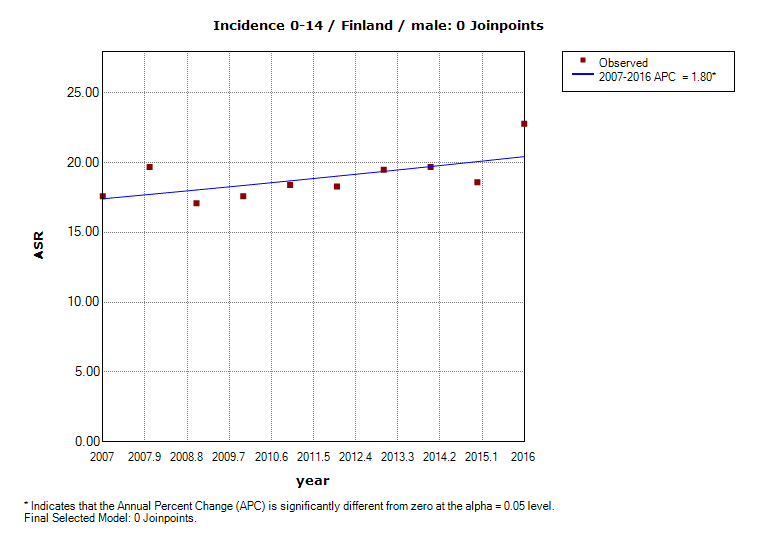 | 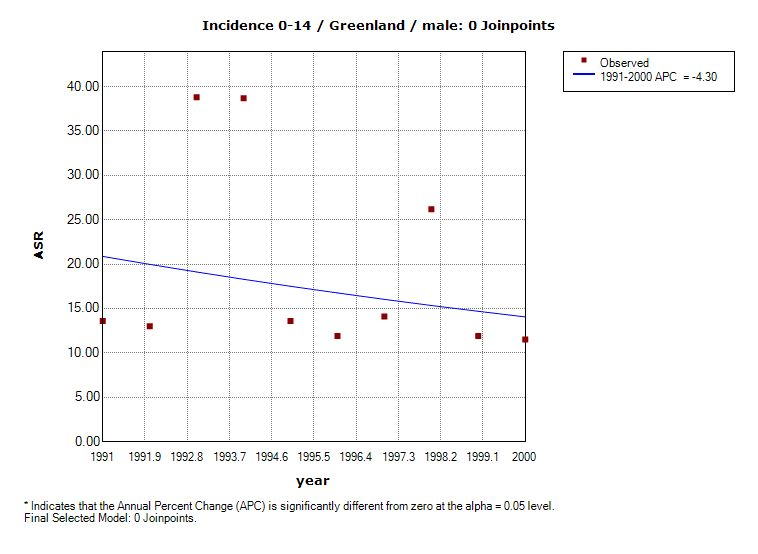 |
| 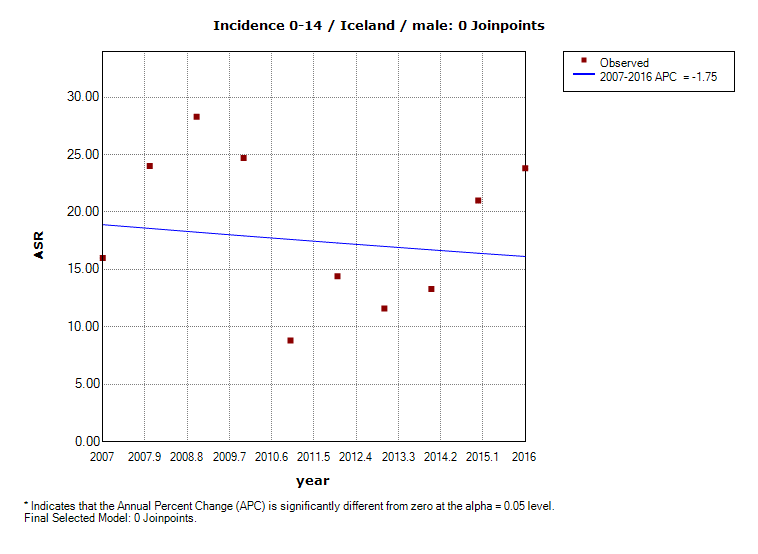 | 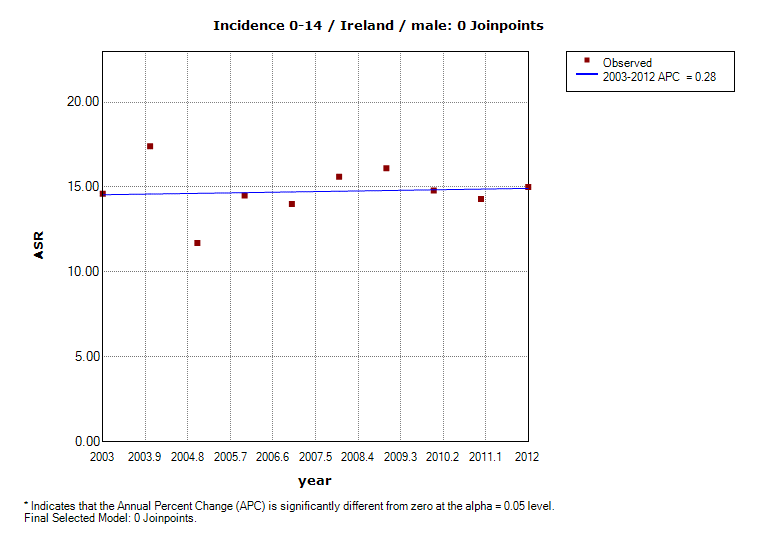 |
| 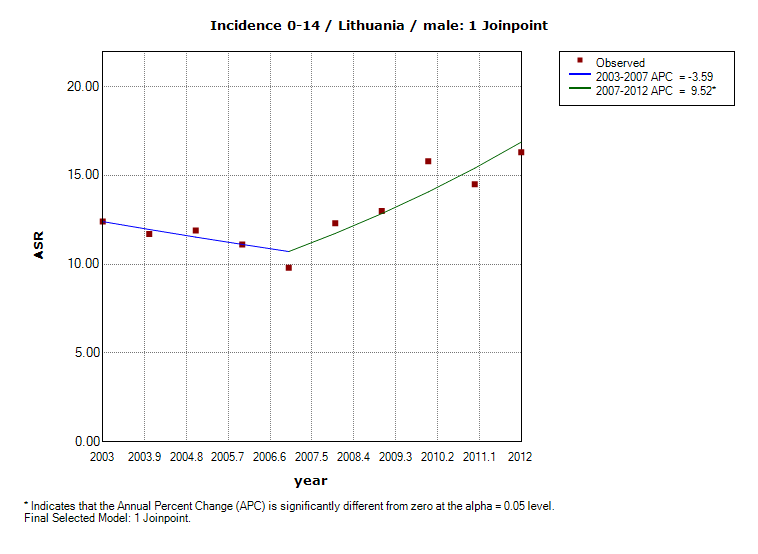 | 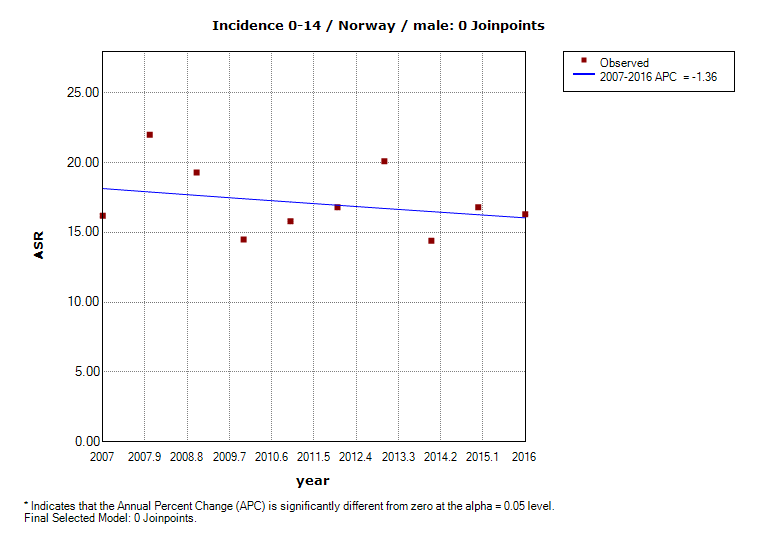 |
| 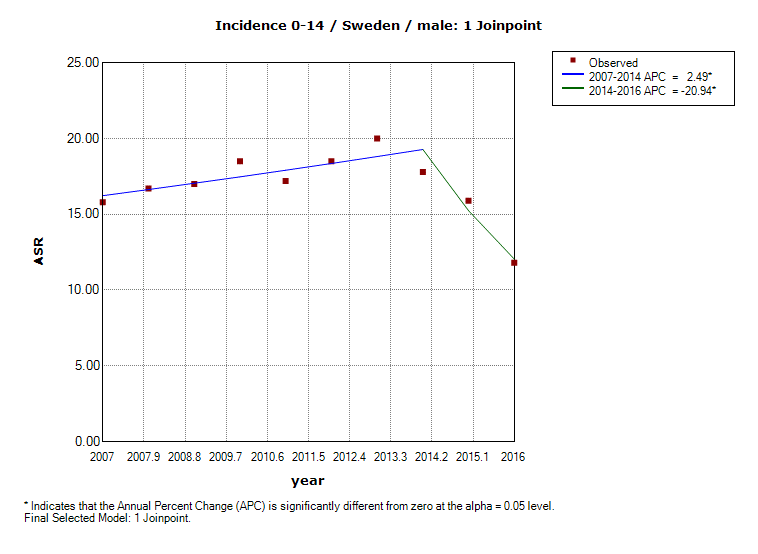 | 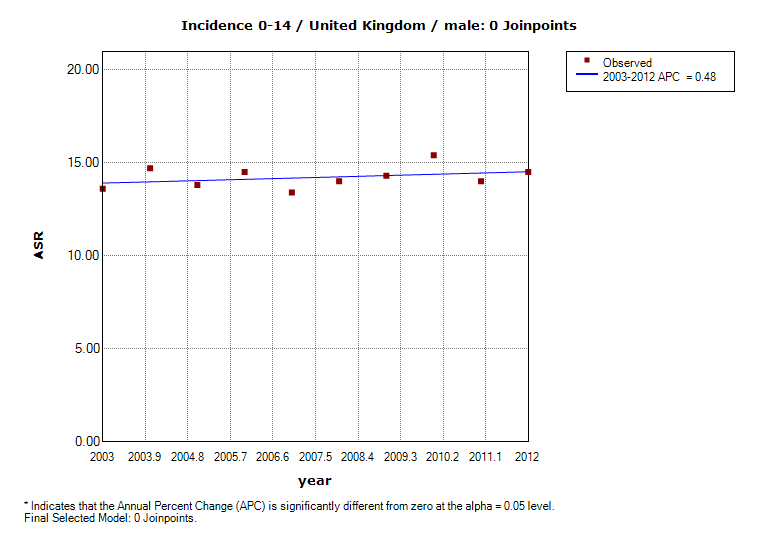 |
| **Western Europe** | |
| 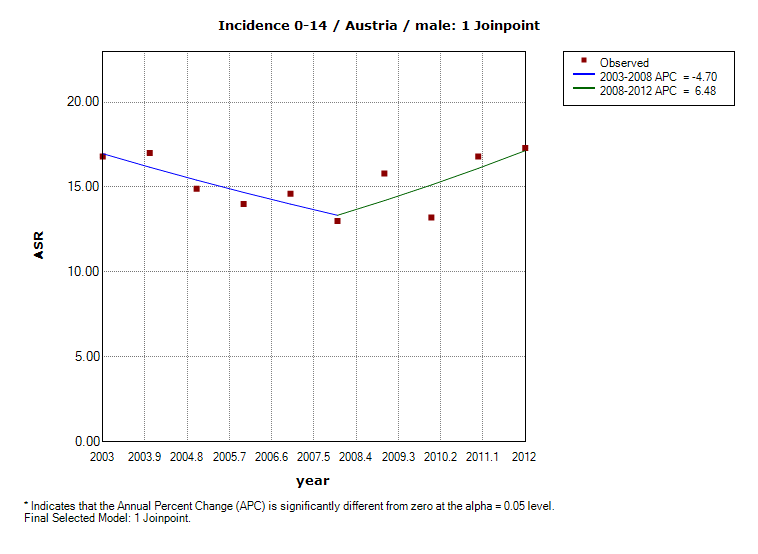 | 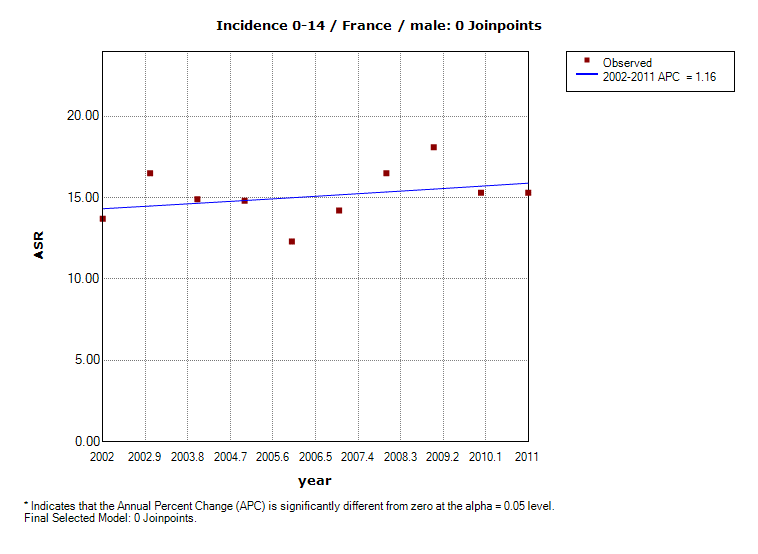 |
| 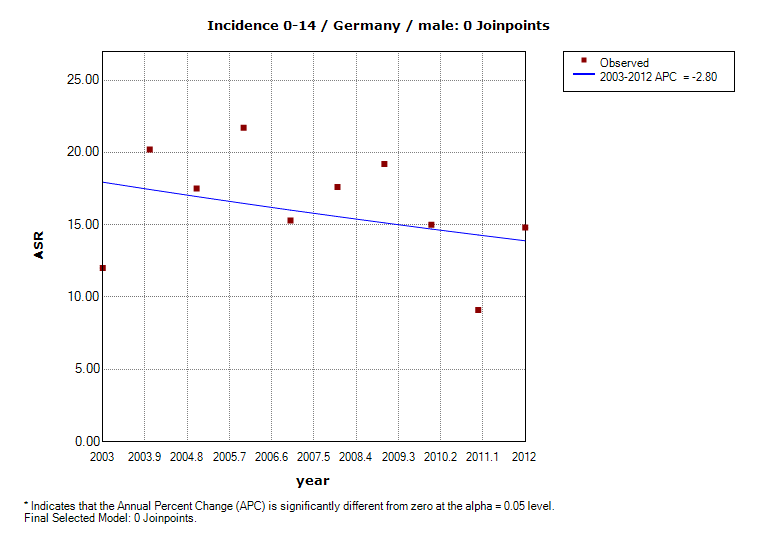 | 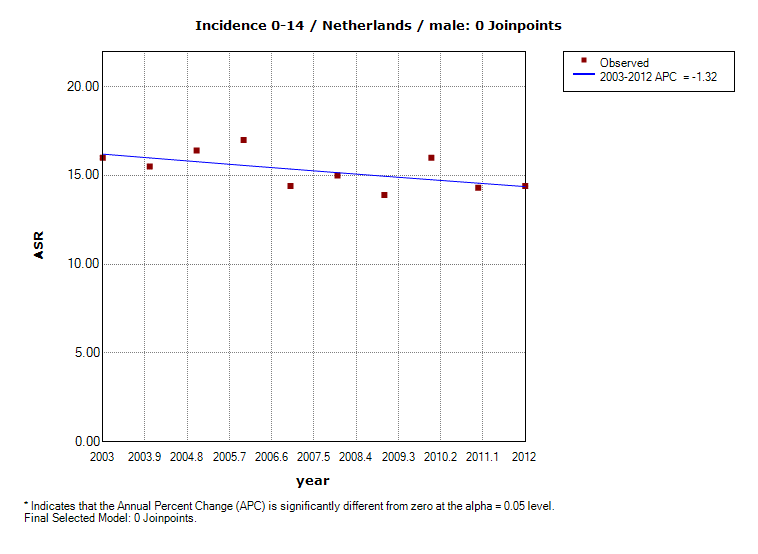 |
| 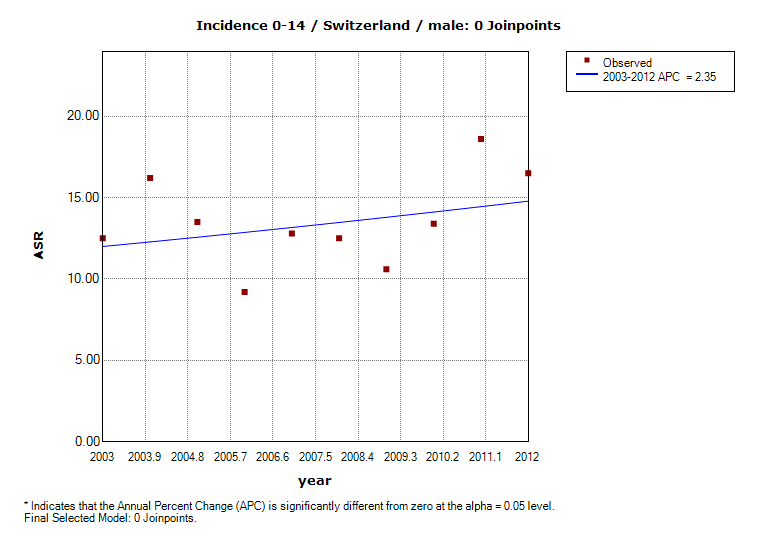 |  |
| **Southern Europe** | |
| 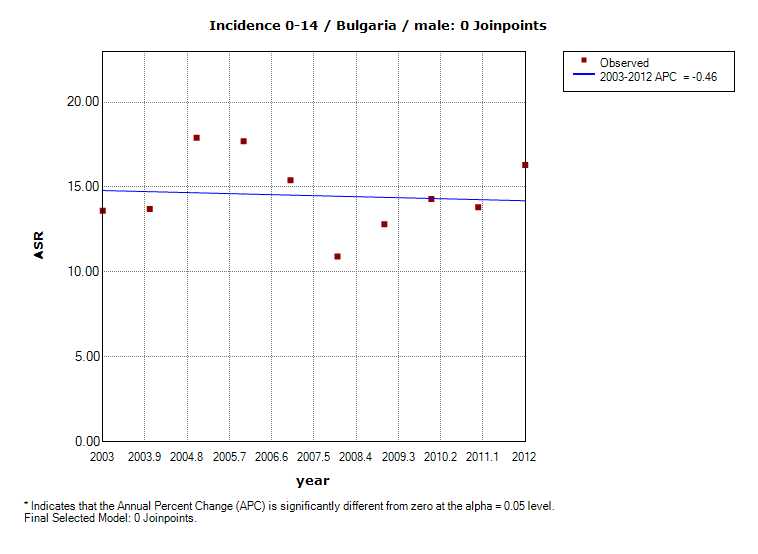 | 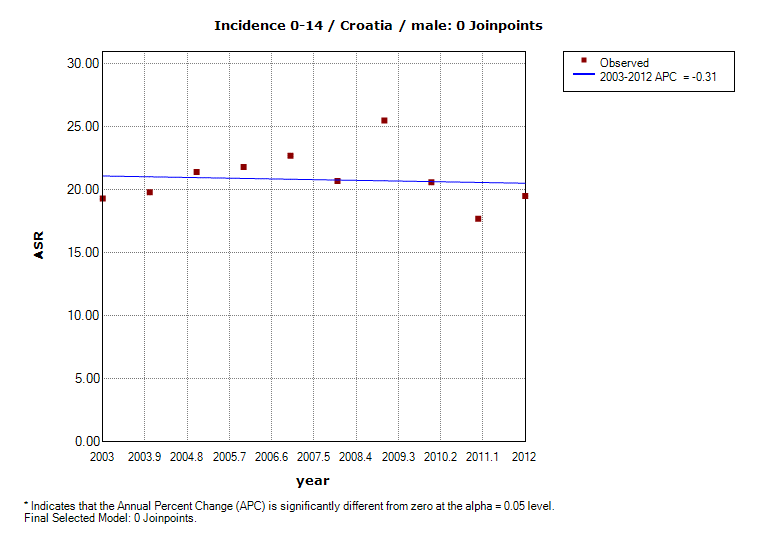 |
| 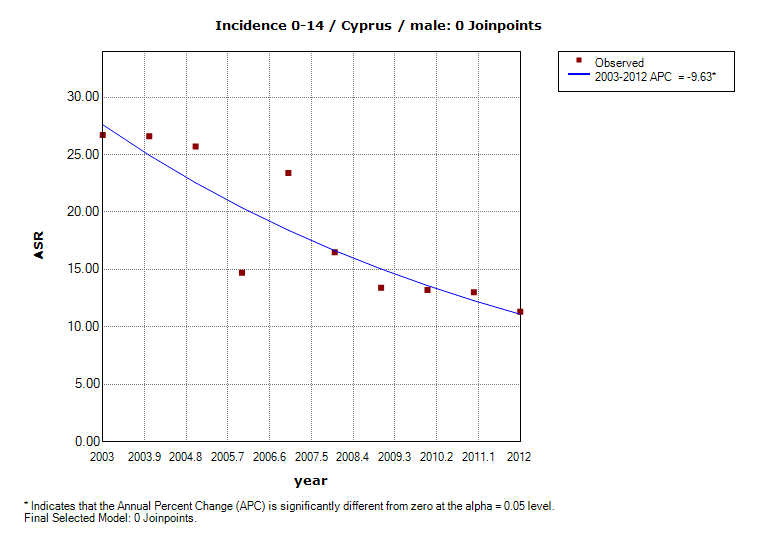 | 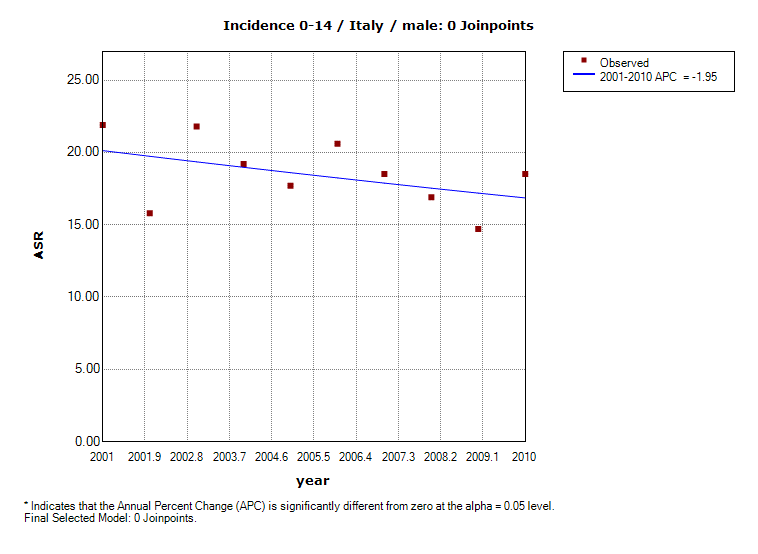 |
| 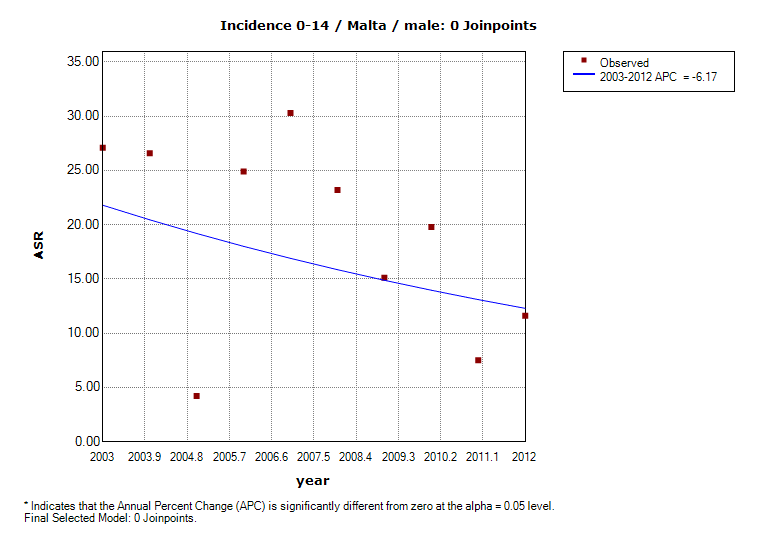 | 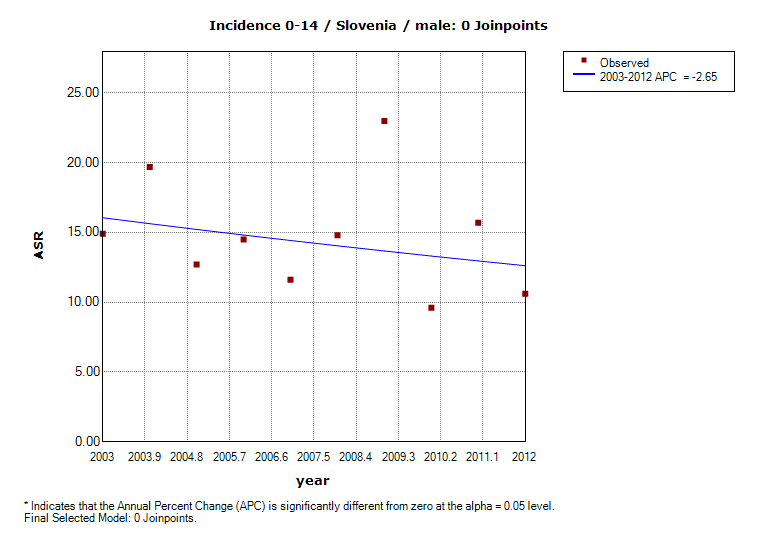 |
| 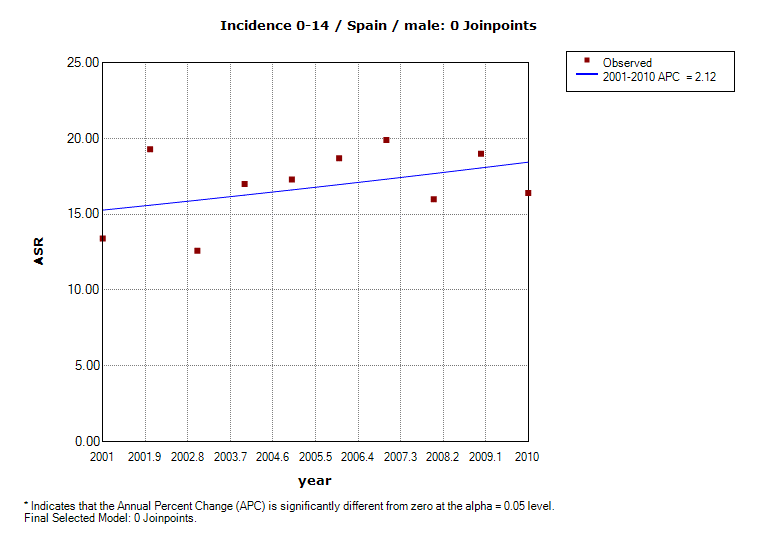 | 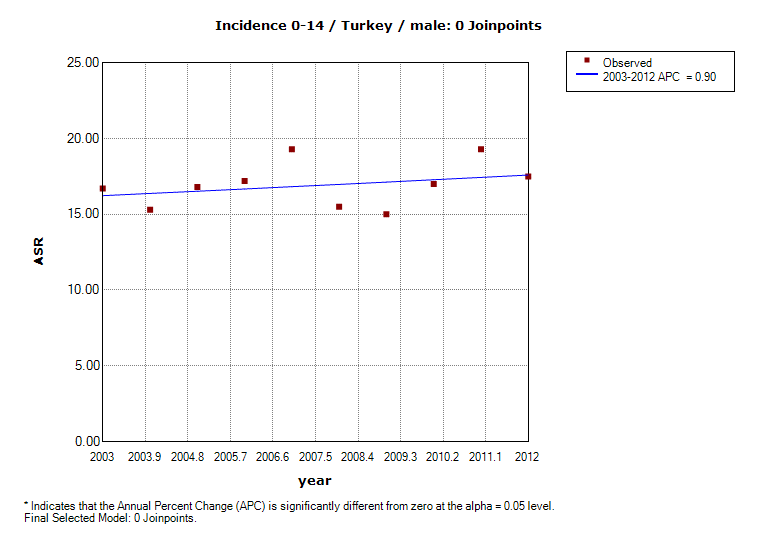 |
| **Eastern Europe** | |
| 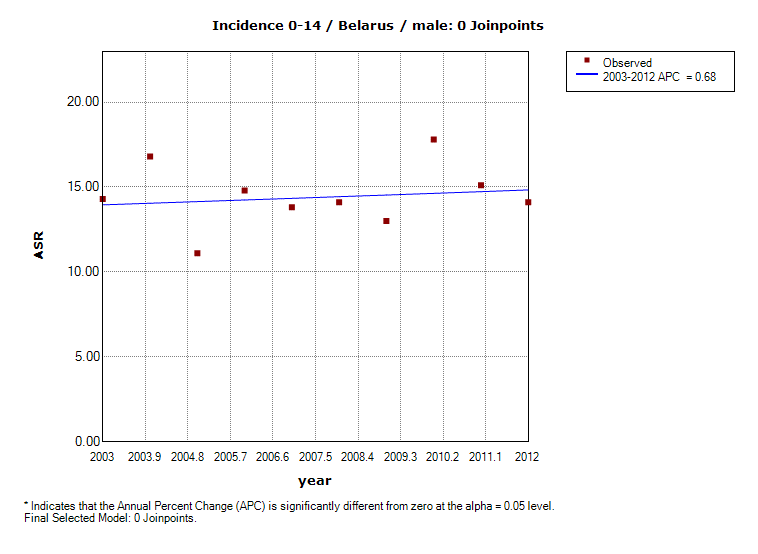 | 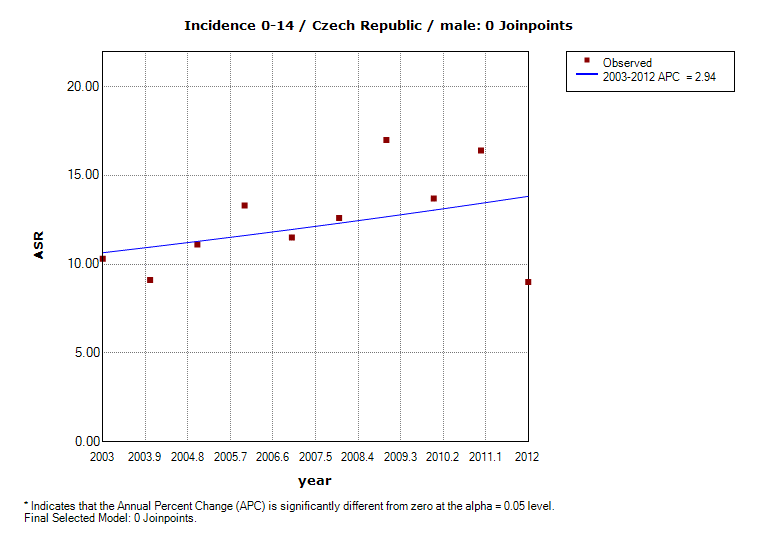 |
| 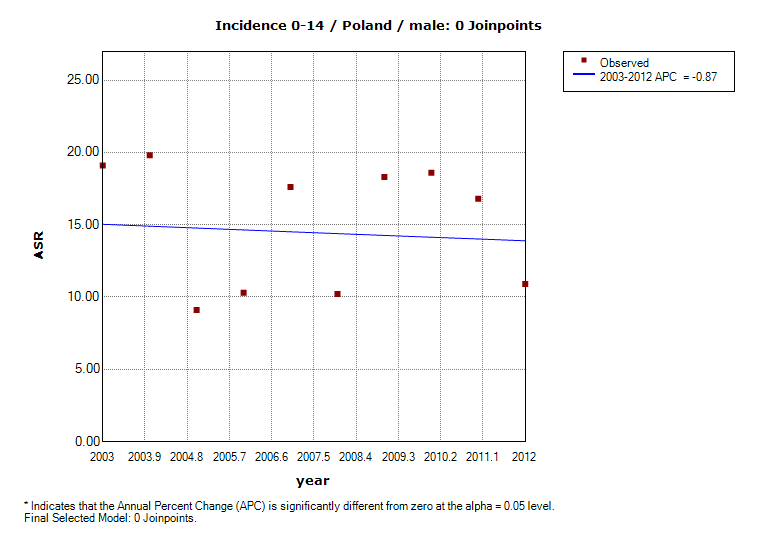 | 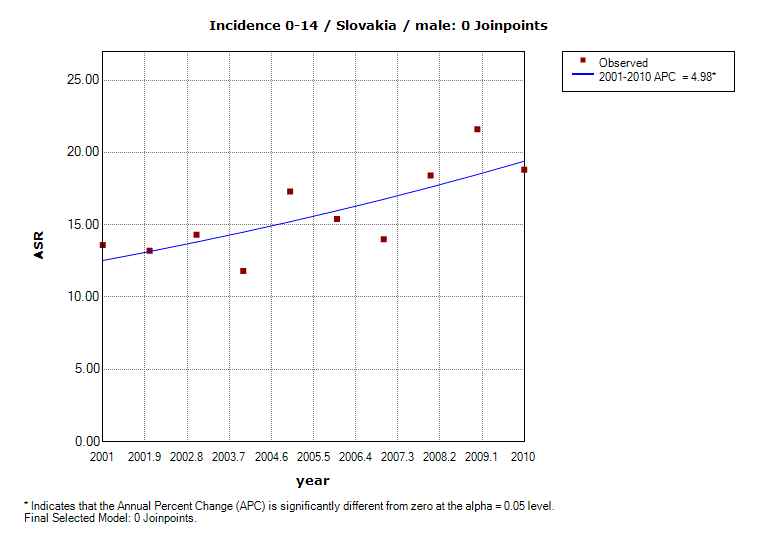 |
| **Africa** | |
| 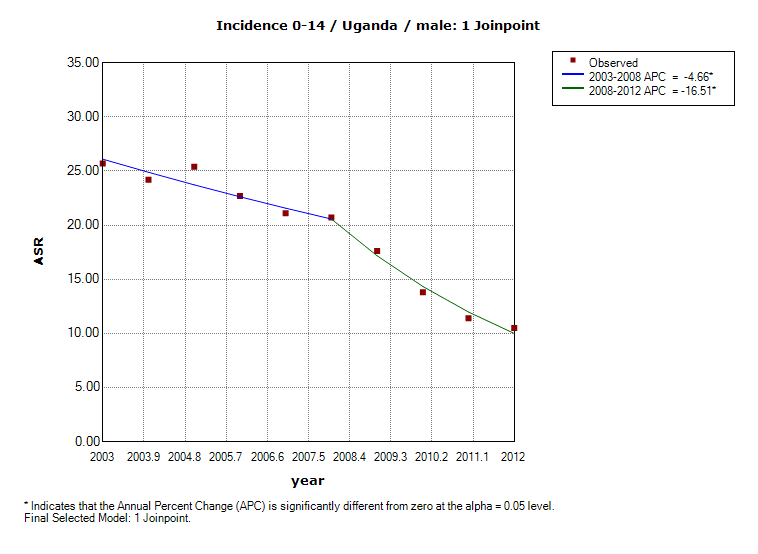 |  |

b.) Incidence female aged 0-14

| **Asia** | |
| --- | --- |
| 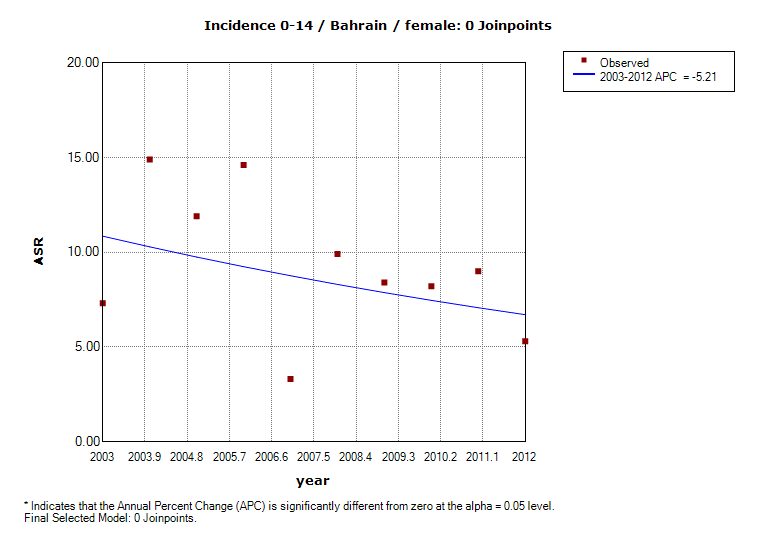 | 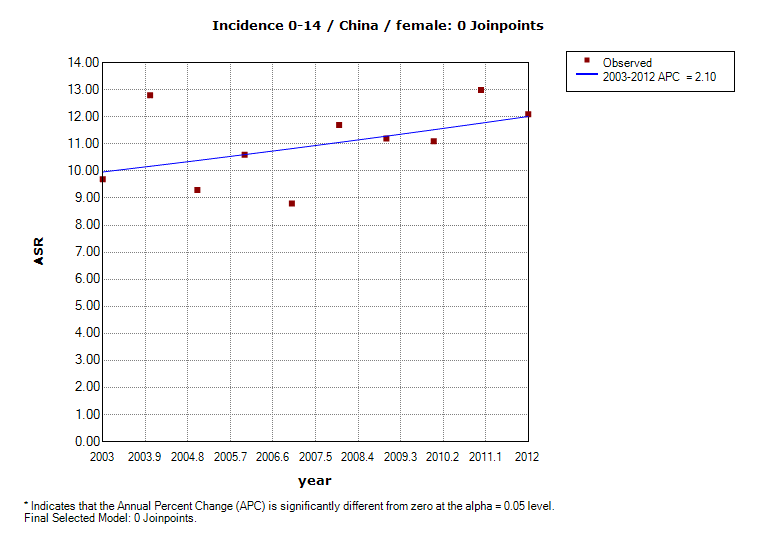 |
| 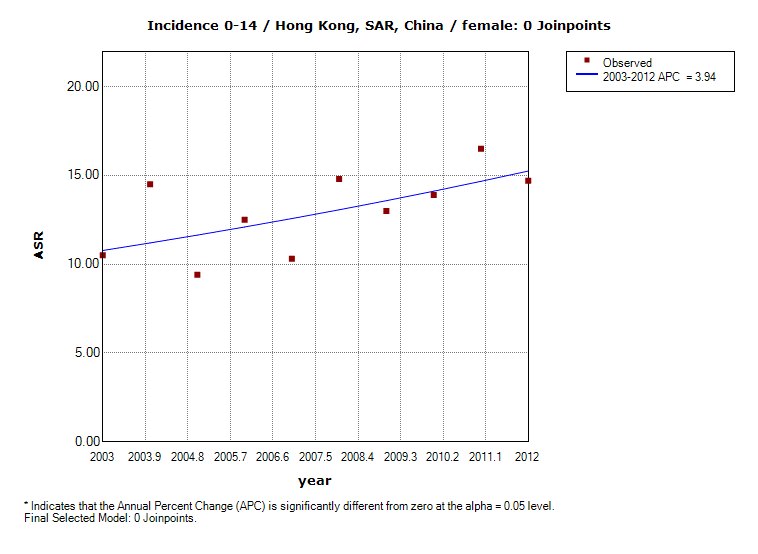 | 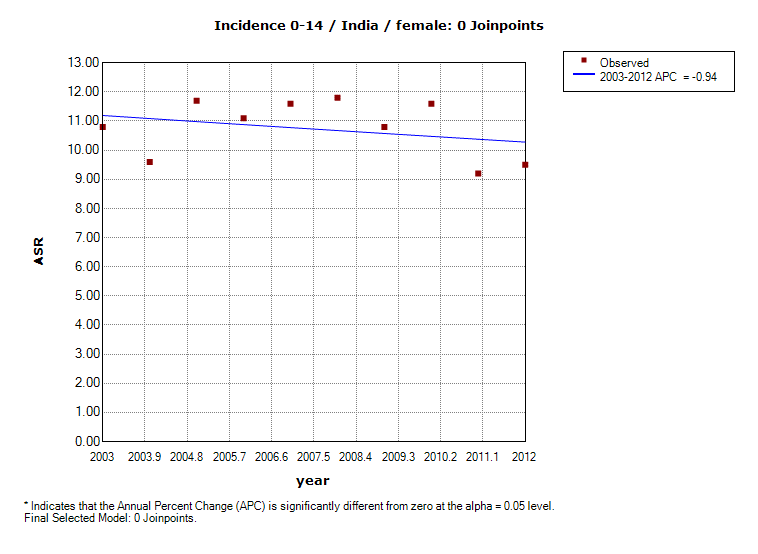 |
| 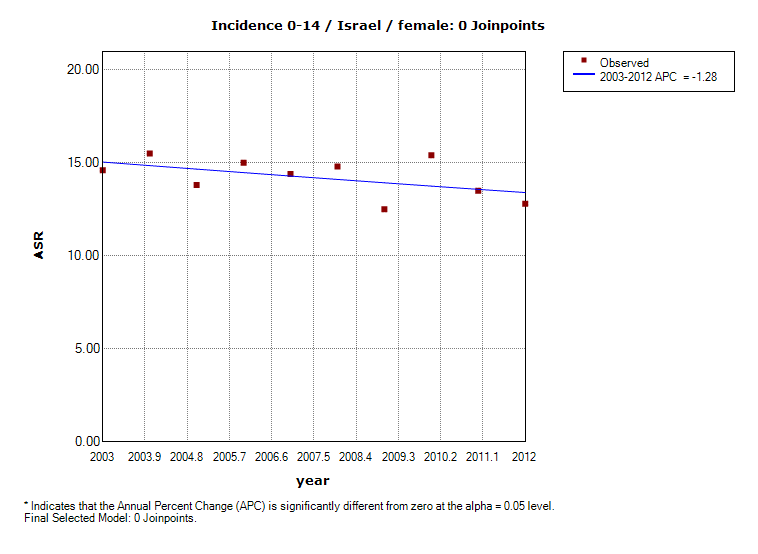 | 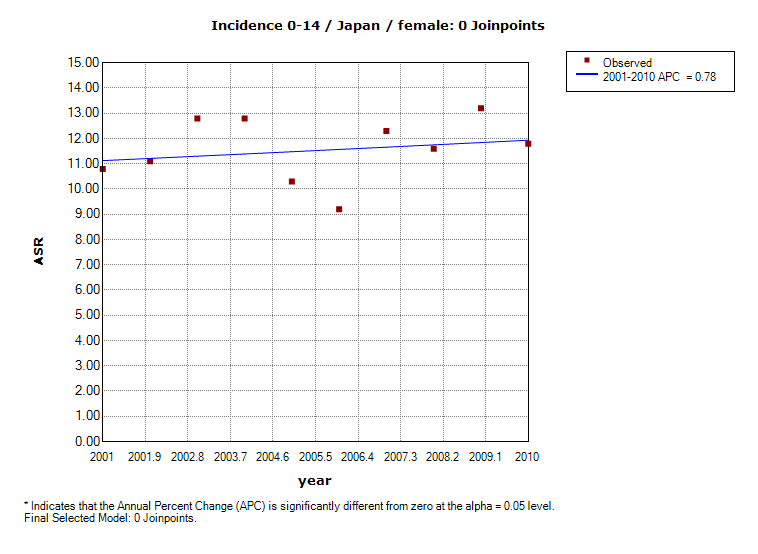 |
| 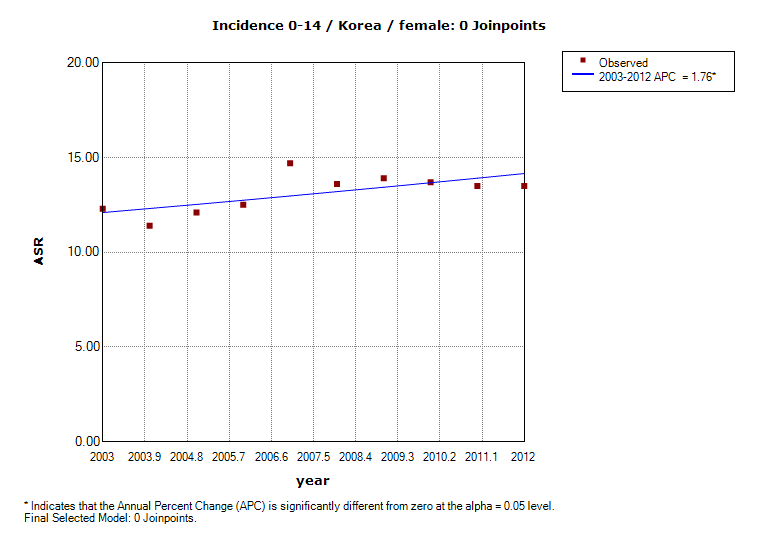 | 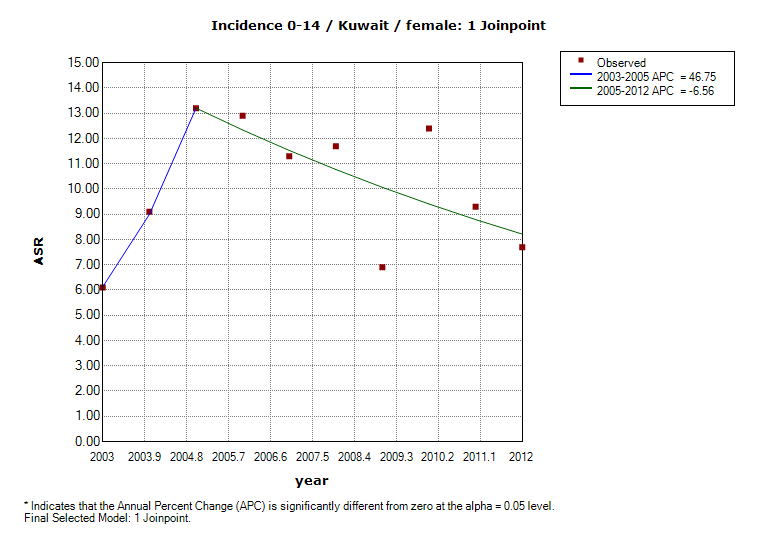 |
| 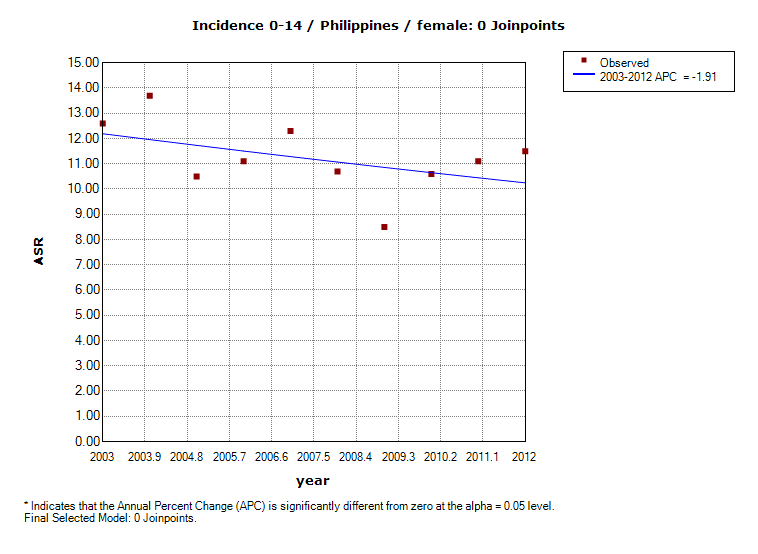 | 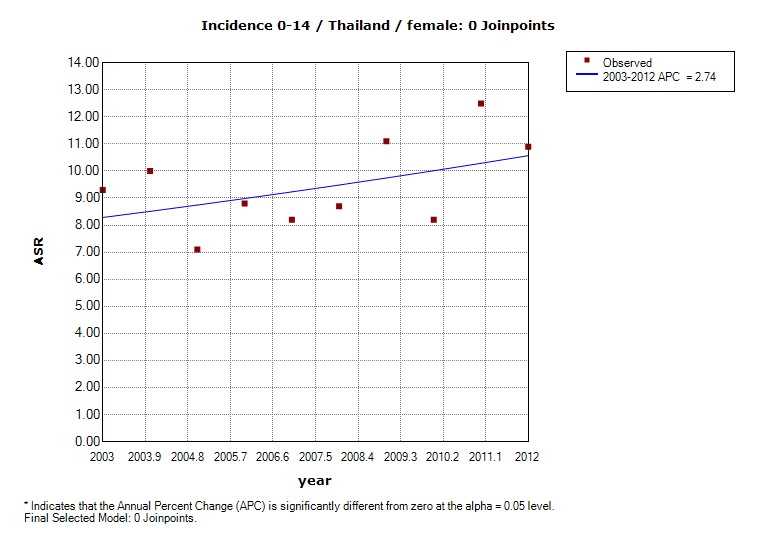 |
| **Oceania** | |
| 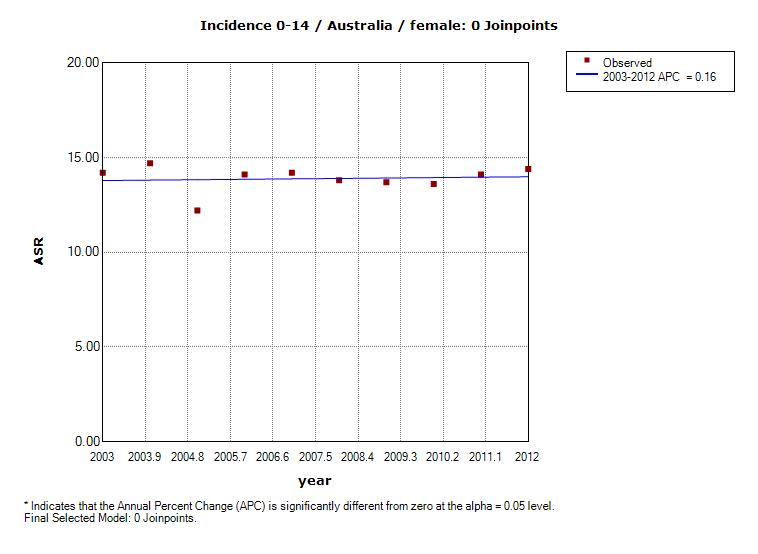 | 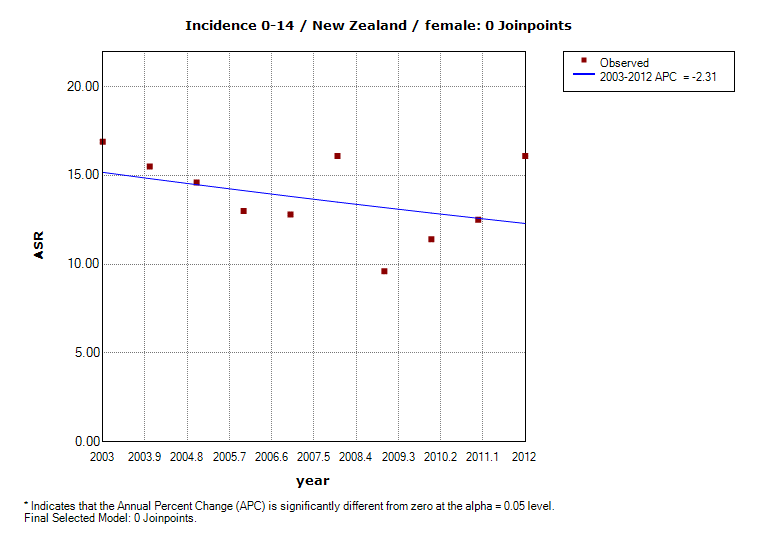 |

| **Northern America** | |
| --- | --- |
| 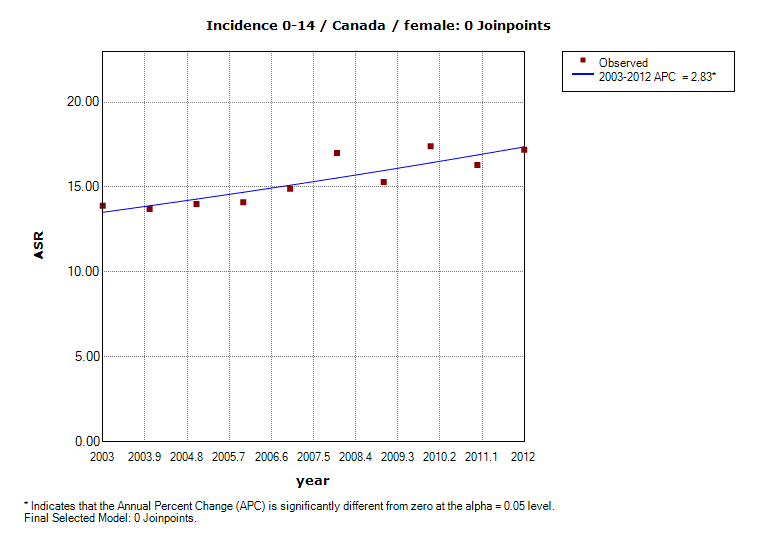 | 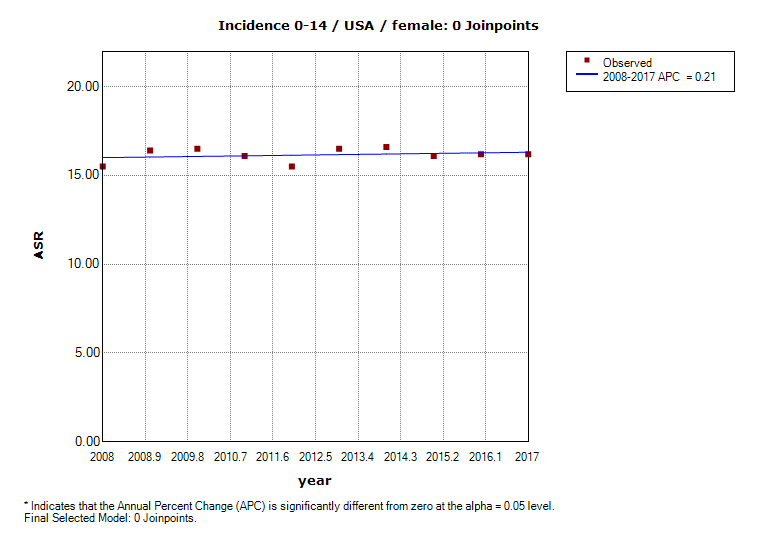 |
| 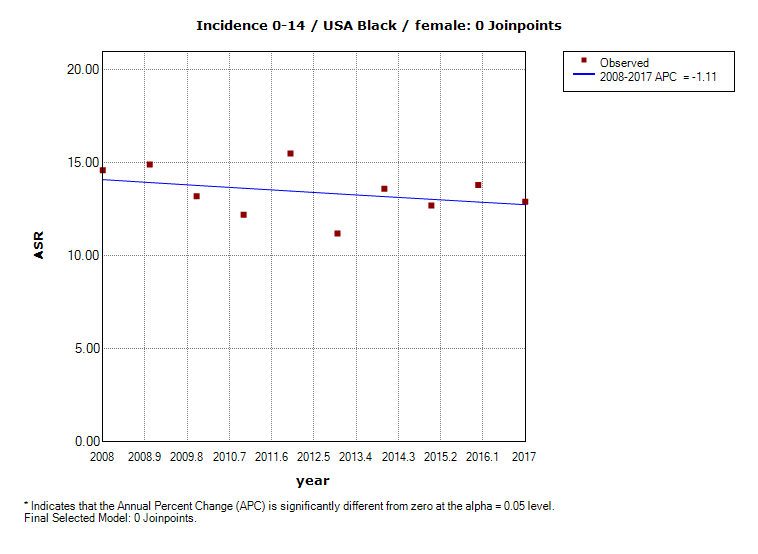 | 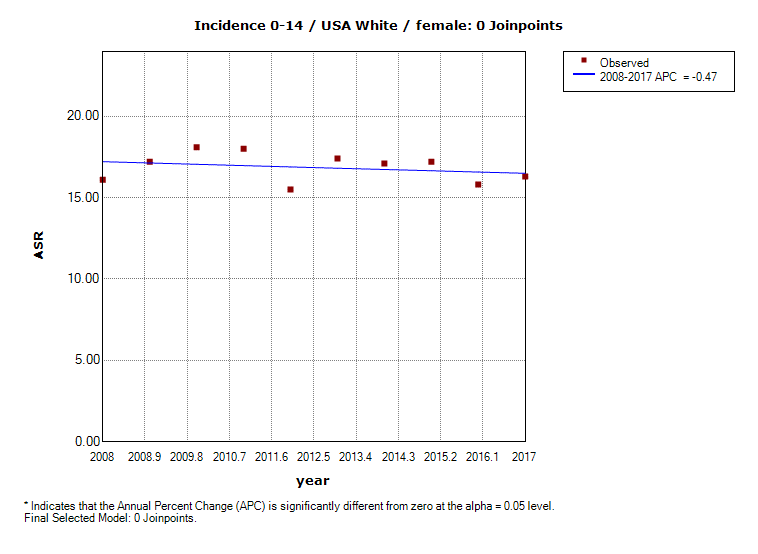 |
| **Southern America** | |
| 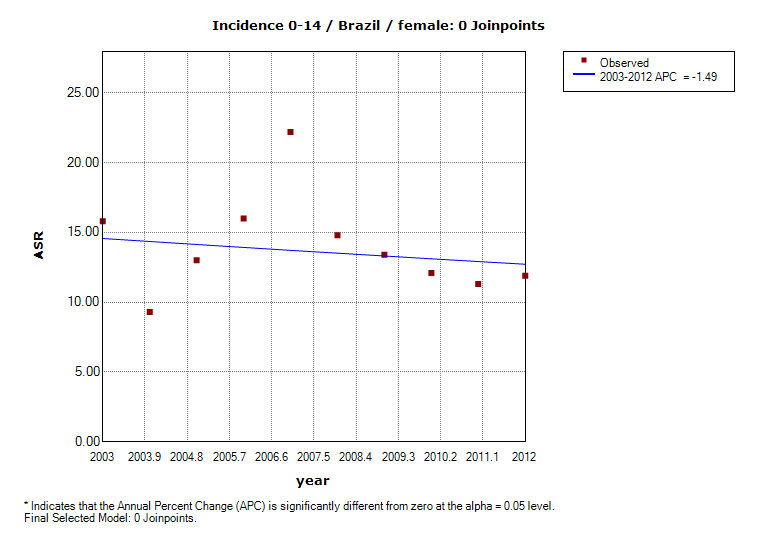 | 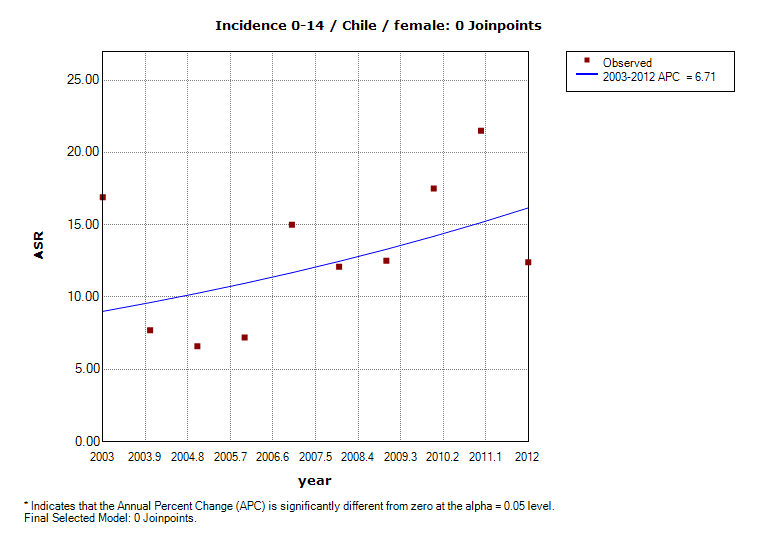 |
| 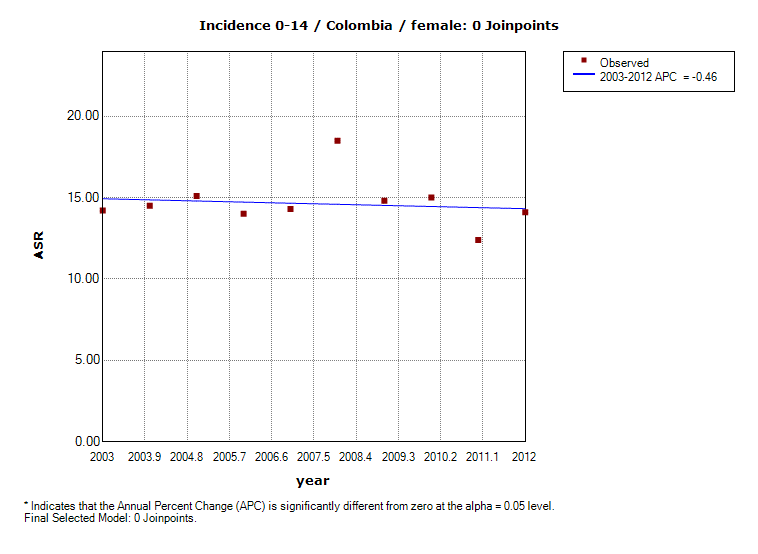 | 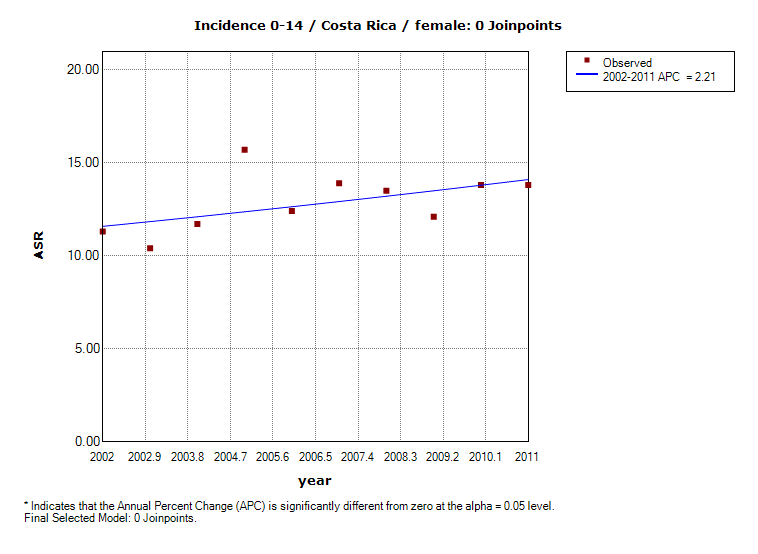 |
| 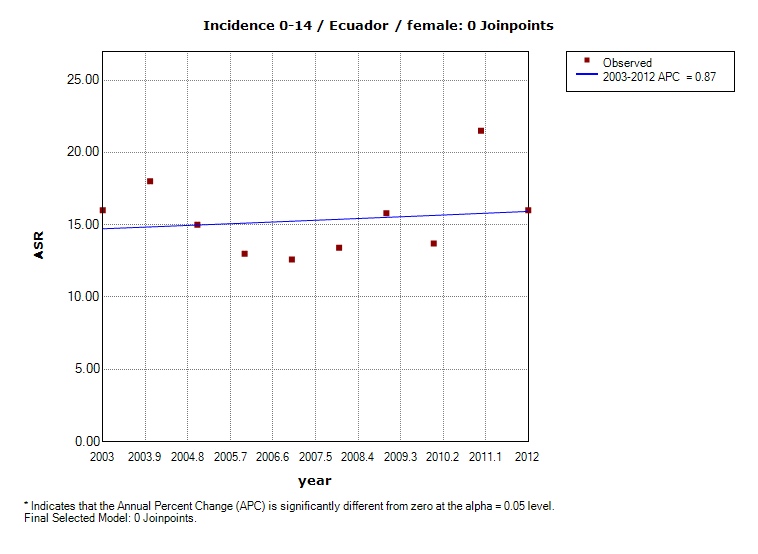 |  |
| **Northern Europe** | |
| 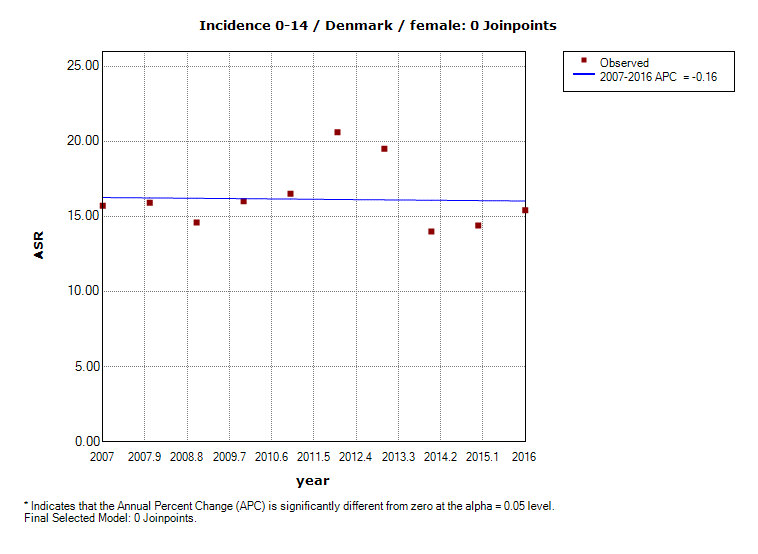 | 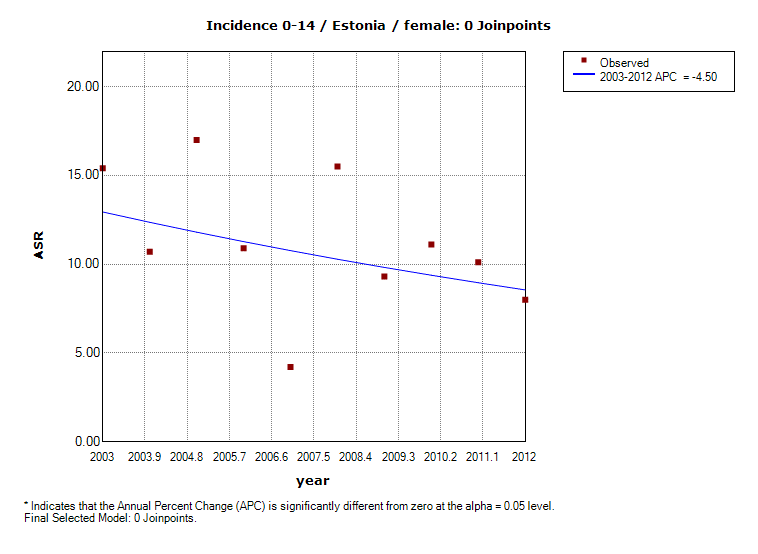 |
| 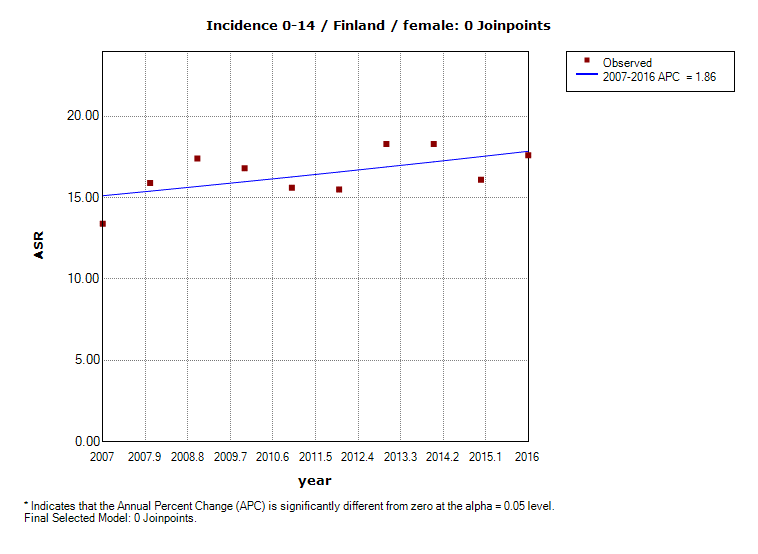 | 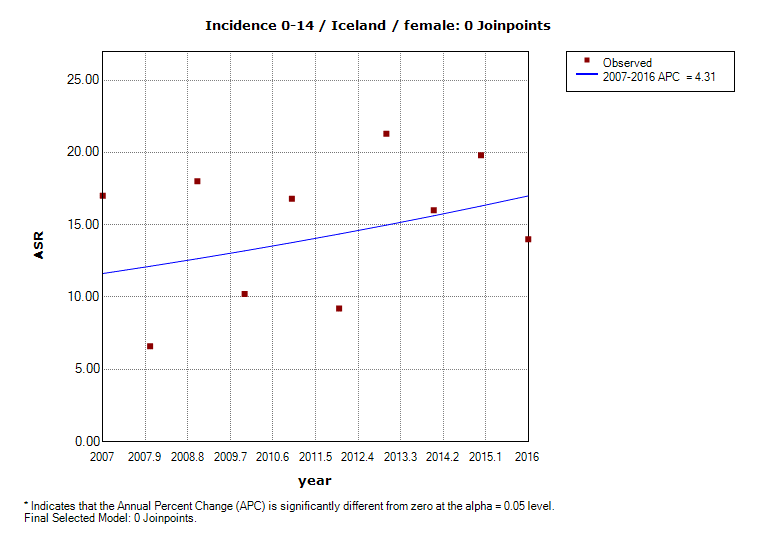 |
| 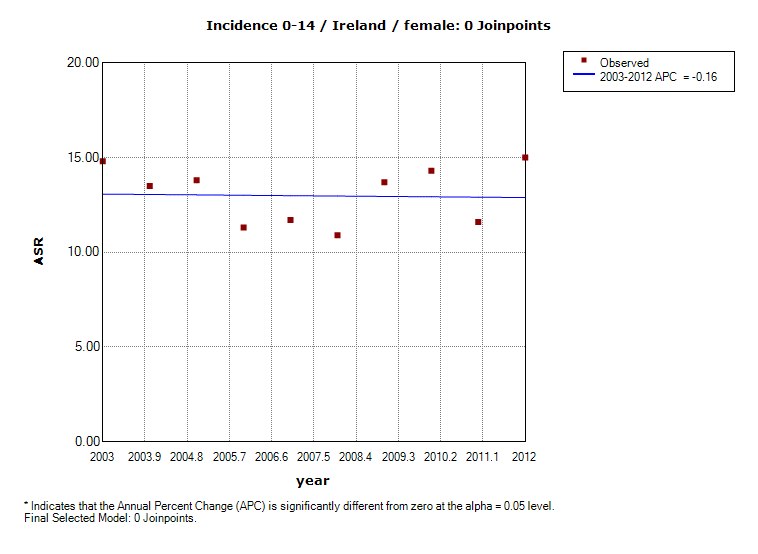 | 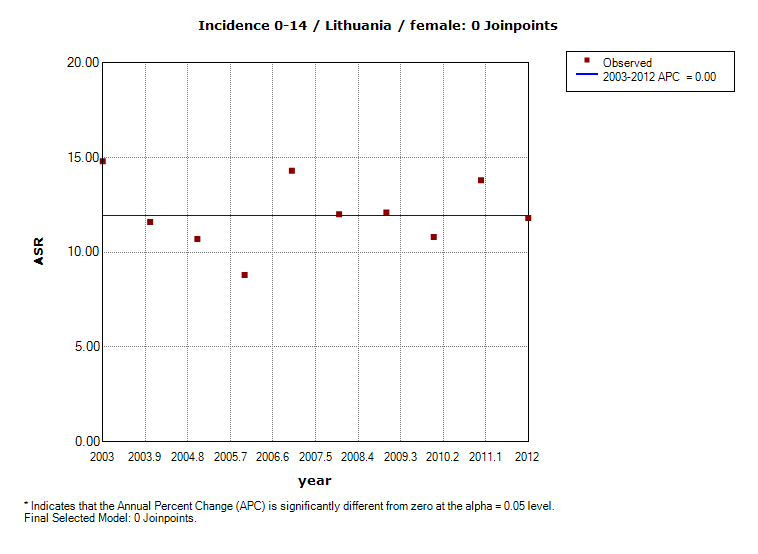 |
| 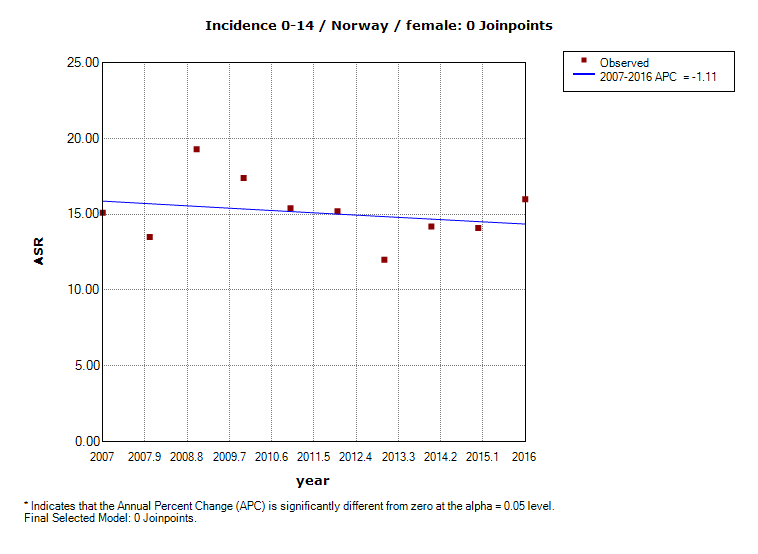 | 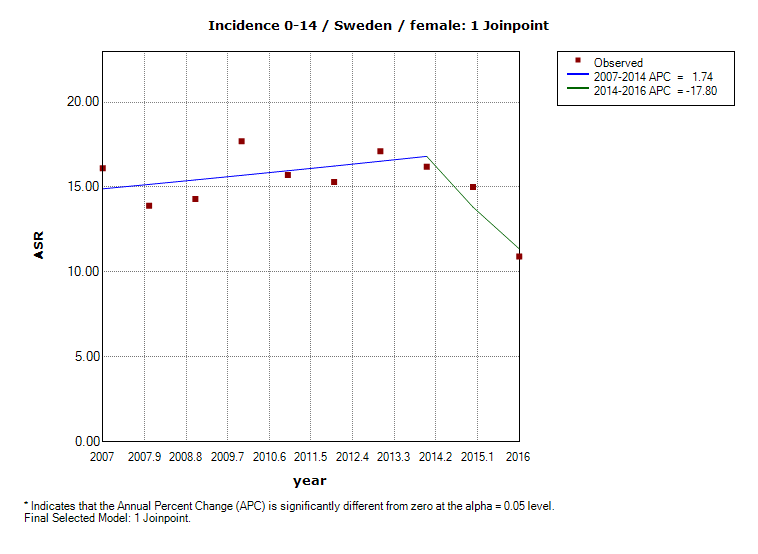 |
| 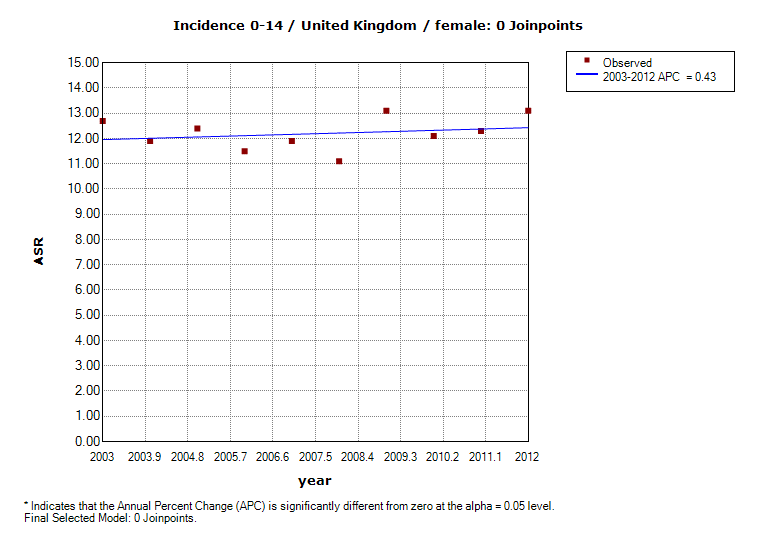 |  |
| **Western Europe** | |
| 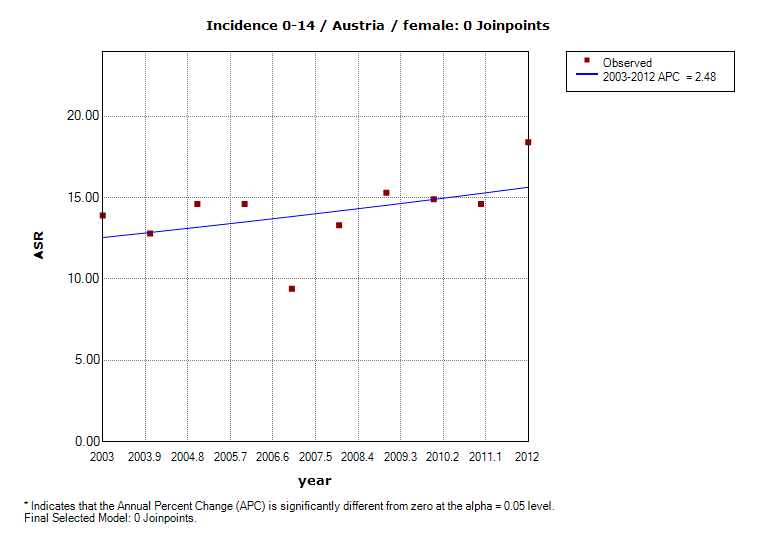 | 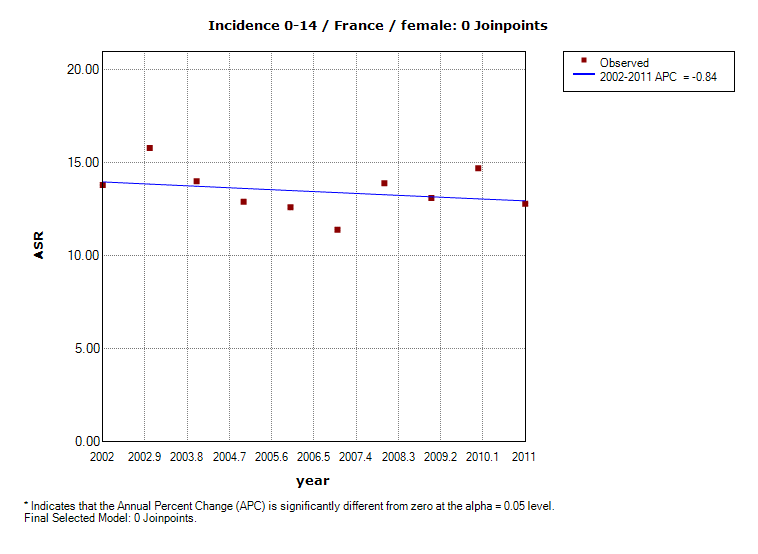 |
| 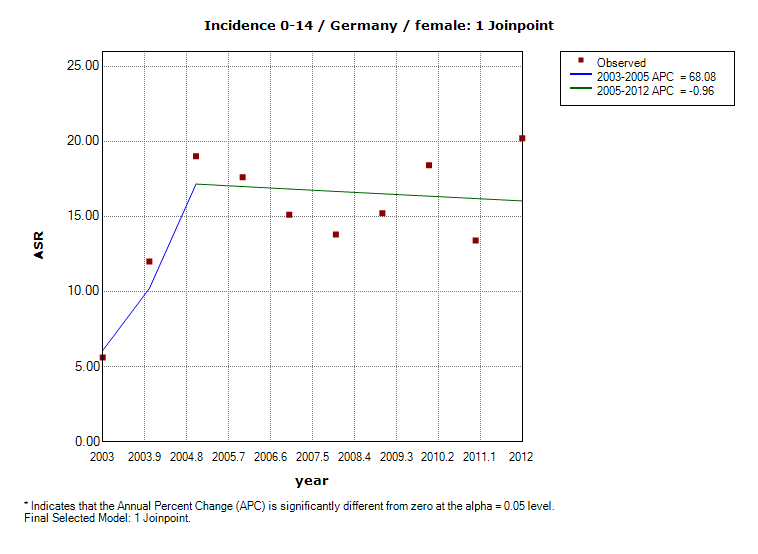 | 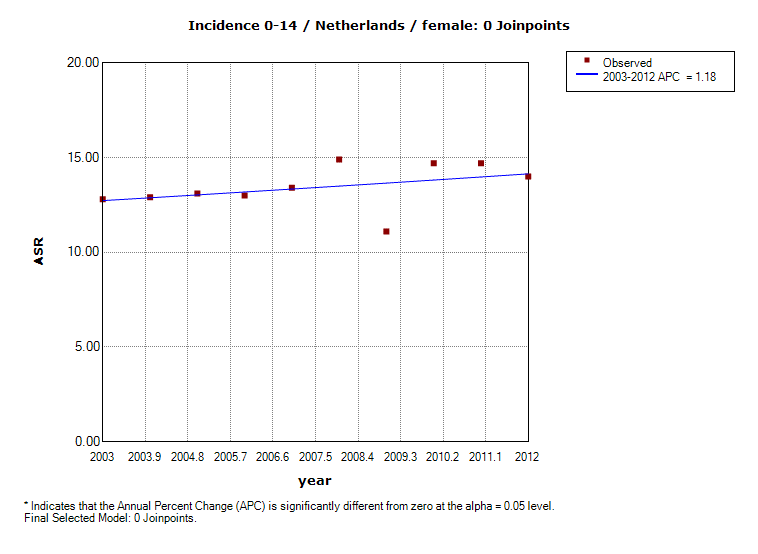 |
| 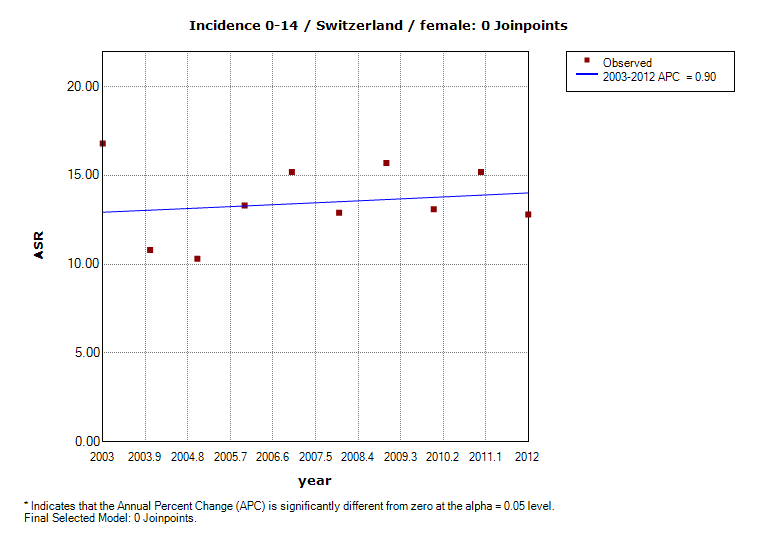 |  |
| **Southern Europe** | |
| 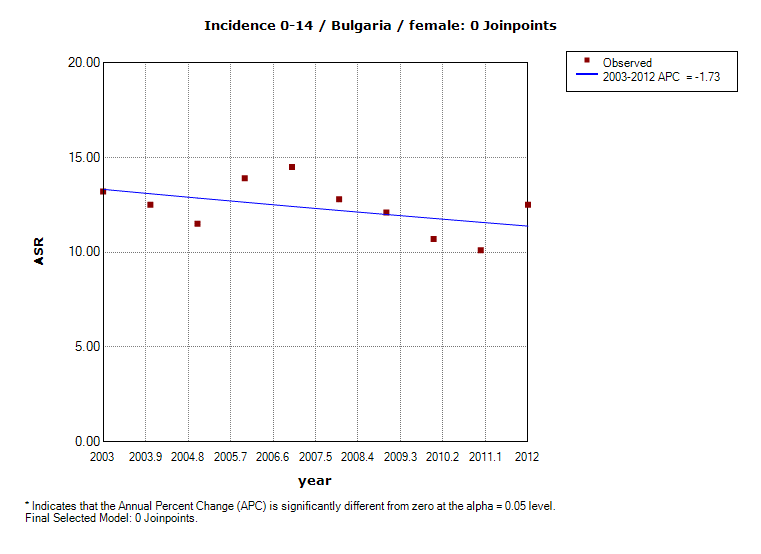 | 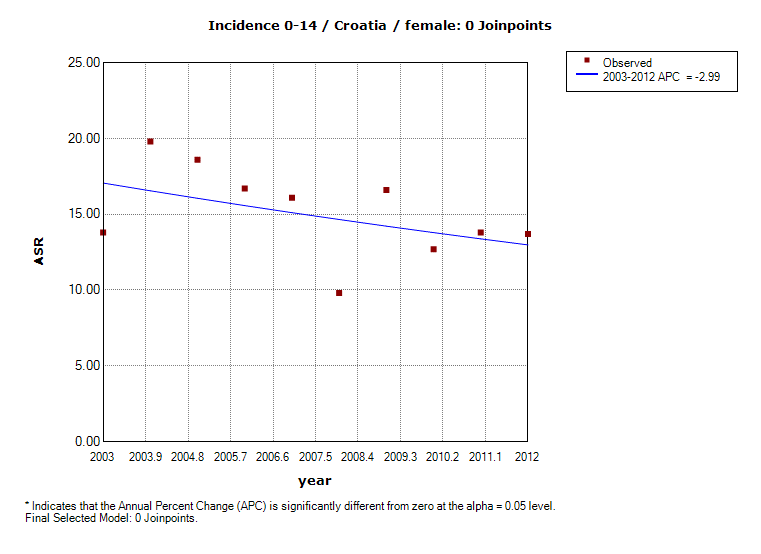 |
| 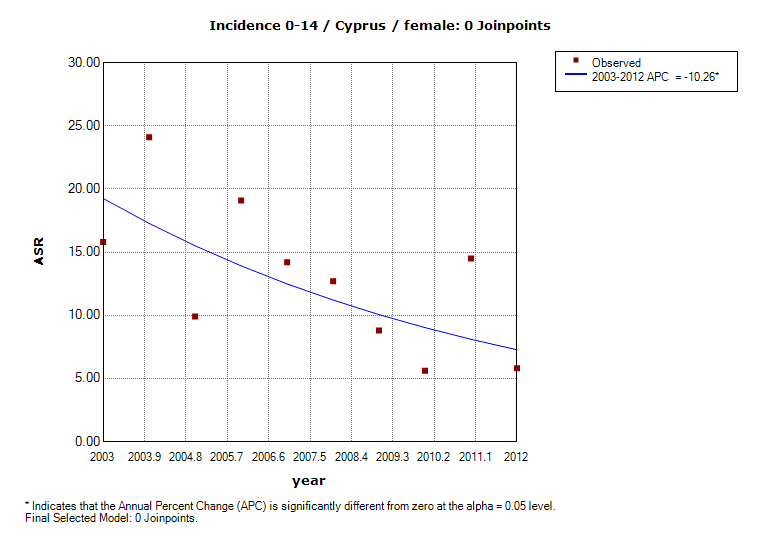 | 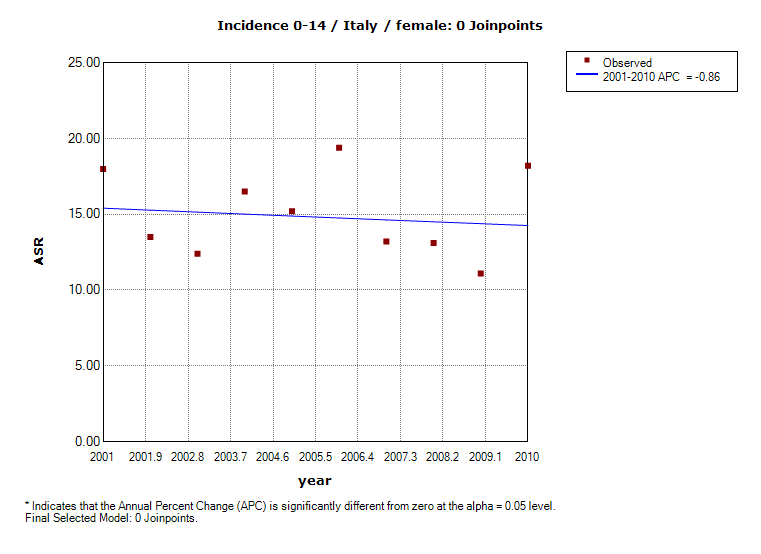 |
| 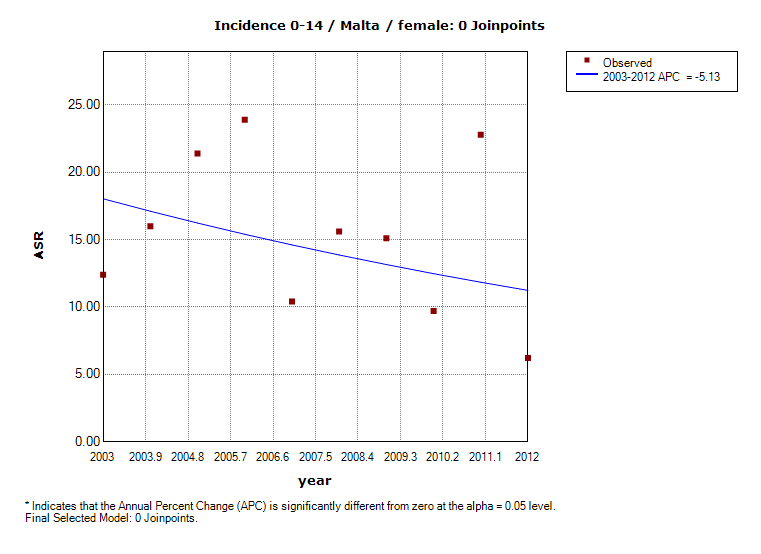 | 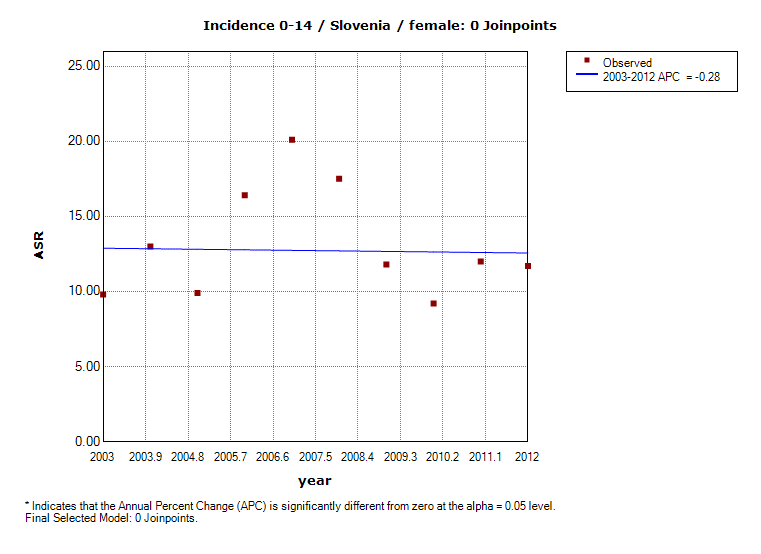 |
| 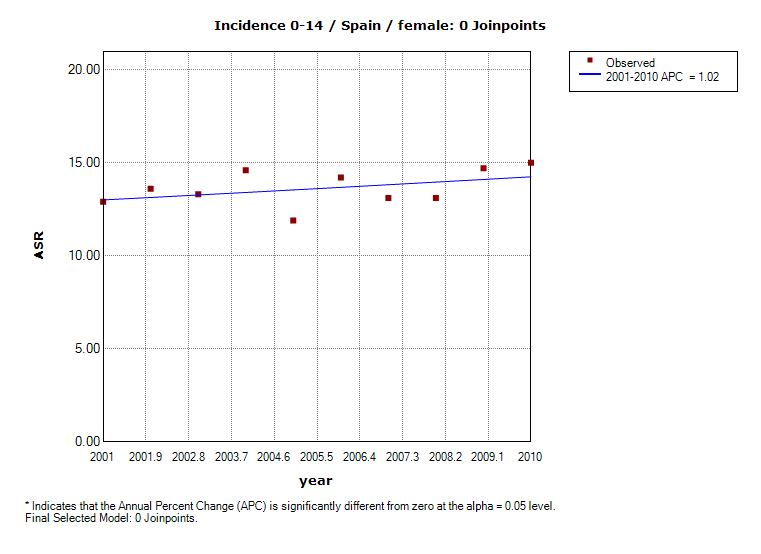 | 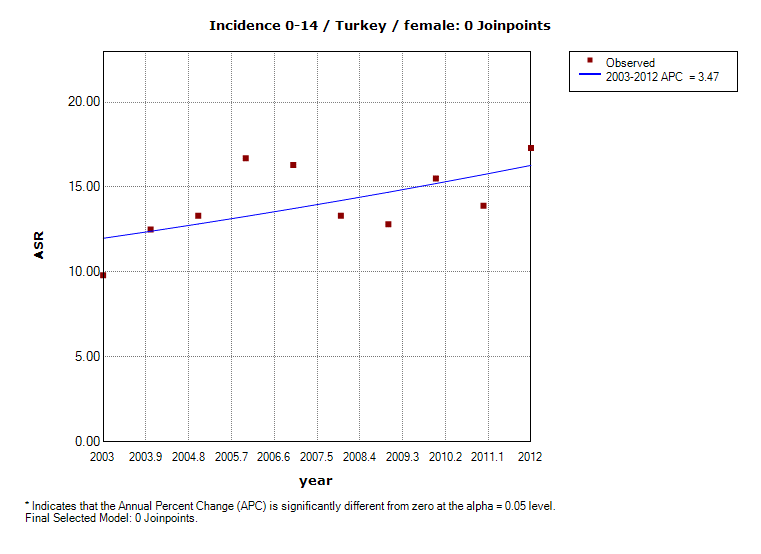 |
| **Eastern Europe** | |
| 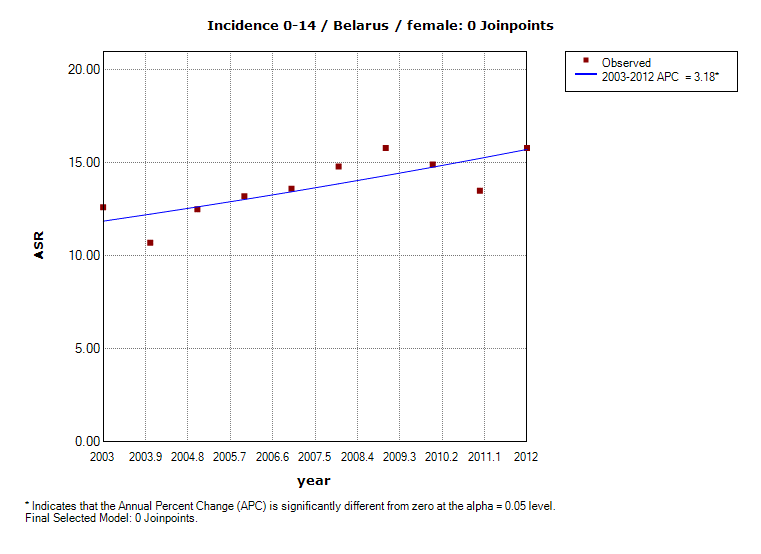 | 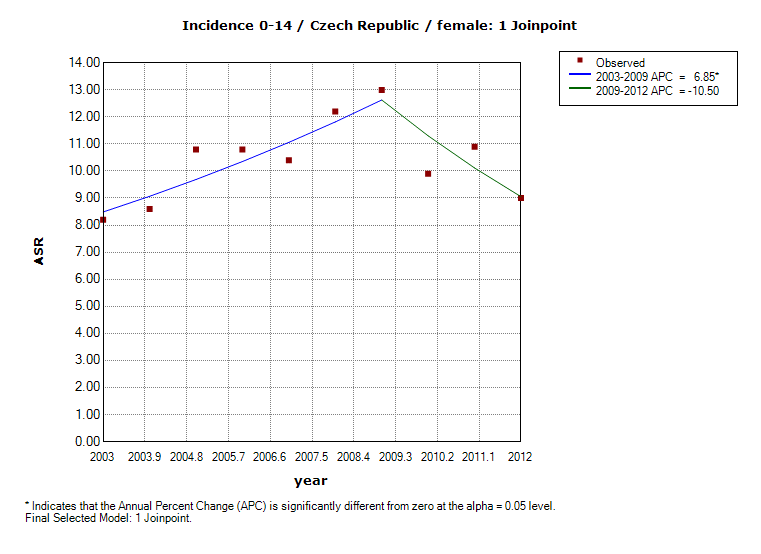 |
| 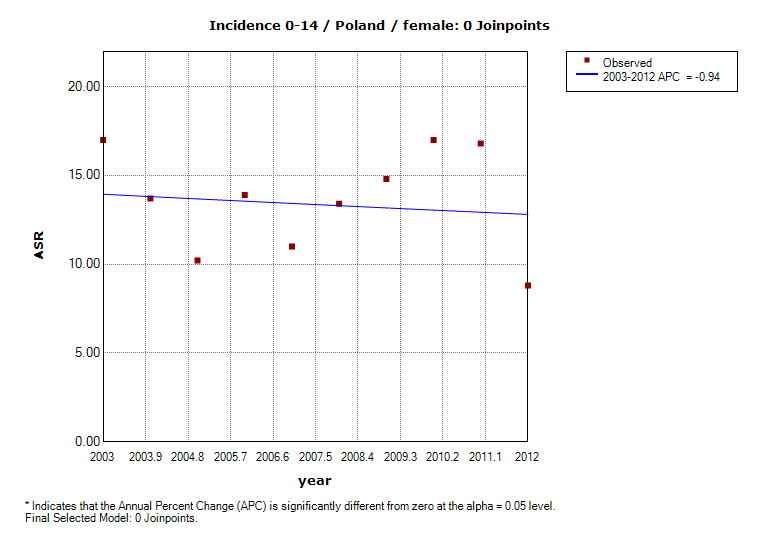 | 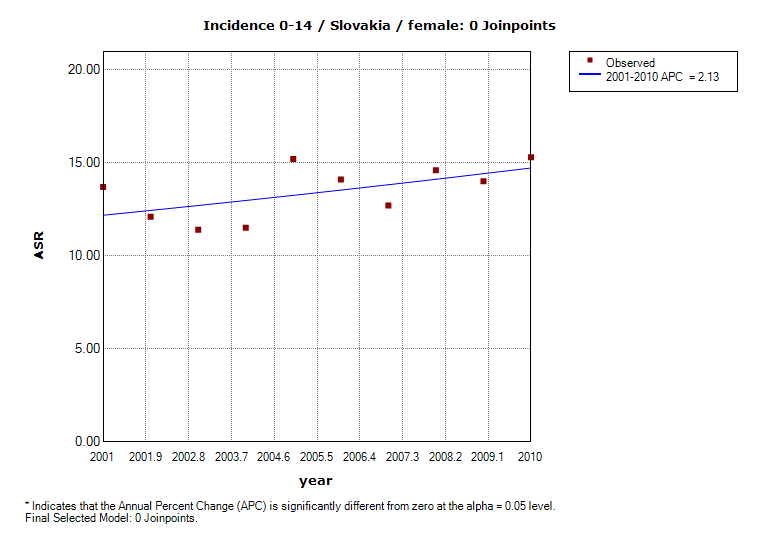 |
| **Africa** | |
| 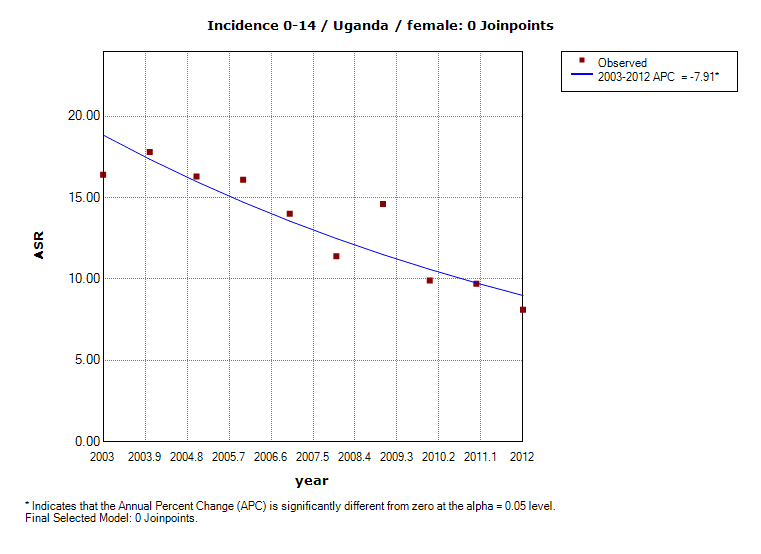 |  |

c.) Mortality male aged 0-14

| **Asia** | |
| --- | --- |
| 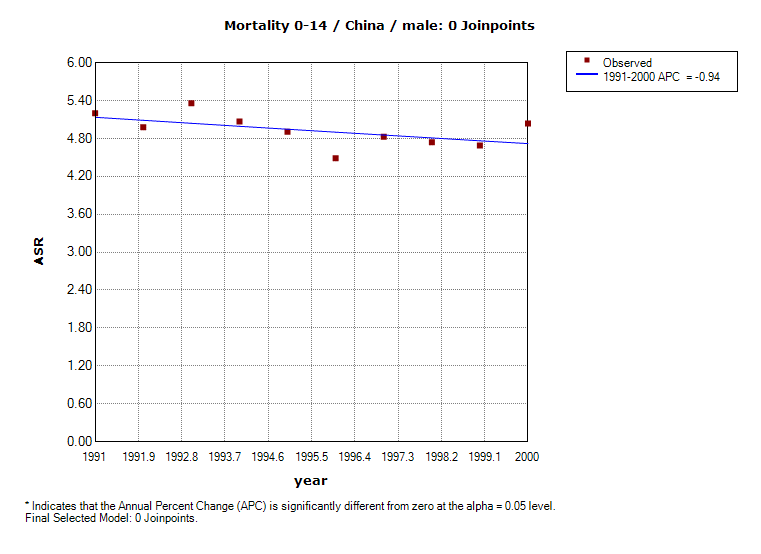 | 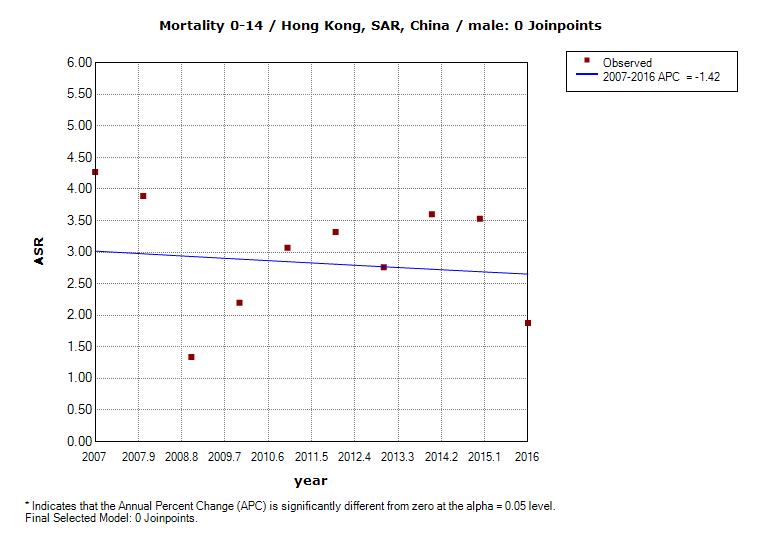 |
| 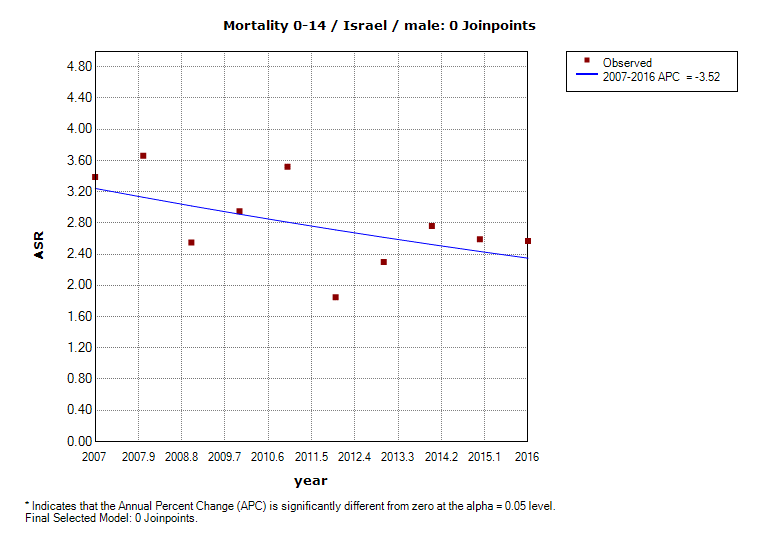 |  |
|  |  |
|  |  |
|  |  |
| **Oceania** | |
|  |  |

| **Northern America** | |
| --- | --- |
|  |  |
|  |  |
| **Southern America** | |
|  |  |
|  |  |
|  |  |
| **Northern Europe** | |
|  |  |
|  |  |
|  |  |
|  |  |
|  |  |
| **Western Europe** | |
|  |  |
|  |  |
|  |  |
| **Southern Europe** | |
|  |  |
|  |  |
|  |  |
|  |  |
| **Eastern Europe** | |
|  |  |
|  |  |
|  |  |

d.) Mortality female aged 0-14

| **Asia** | |
| --- | --- |
|  |  |
|  |  |
|  |  |
|  |  |
|  |  |
| **Oceania** | |
|  |  |

| **Northern America** | |
| --- | --- |
|  |  |
|  |  |
| **Southern America** | |
|  |  |
|  |  |
|  |  |
| **Northern Europe** | |
|  |  |
|  |  |
|  |  |
|  |  |
|  |  |
| **Western Europe** | |
|  |  |
|  |  |
|  |  |
| **Southern Europe** | |
|  |  |
|  |  |
|  |  |
|  |  |
| **Eastern Europe** | |
|  |  |
|  |  |
|  |  |
